# Cardiovascular Disease Meets Cancer: Exploring the Epidemiology in China and Homotherapy Targeting Intersectional Mechanisms

**DOI:** 10.1002/advs.202501820

**Published:** 2025-08-30

**Authors:** Shihan Xiang, Yujie Shan, Shihong Xu, Jing Wang, Meng Luo, Jiaojiao Zhou, Qishan Chen

**Affiliations:** ^1^ Institute for Developmental and Regenerative Cardiovascular Medicine Department of Cardiology Xinhua Hospital Affiliated with Shanghai Jiaotong University School of Medicine Shanghai 202150 China; ^2^ Zhejiang University School of Medicine Hangzhou 310058 China; ^3^ The Key Laboratory of Cancer Prevention and Intervention China National Ministry of Education The Second Affiliated Hospital Zhejiang University School of Medicine Hangzhou 310000 China; ^4^ Department of Plastic Surgery the Second Affiliated Hospital Zhejiang University School of Medicine Hangzhou 310000 China

**Keywords:** cancer, cardiovascular disease, cardio‐oncology, homotherapy

## Abstract

Cardiovascular disease (CVD) and cancer currently rank among the leading causes of mortality worldwide. Epidemiological studies reveal considerable overlap between CVD and cancer populations, posing challenges in the development of common clinical interventions. This review integrates global perspectives on the shared burden of CVD and cancer, with a focused analysis of regional and demographic disparities within China, as well as significant geographic and lifestyle diversity. This review explores preventive and therapeutic strategies by examining shared risk factors and intersectional mechanisms. First, as the easiest and most effective way, lifestyle modifications that benefit both CVD and cancer are summarized, including physical activity, diet modification, sleep pattern changes, and a reduction in tobacco and alcohol use. Key mechanistic intersections between CVD and cancer, such as metabolic regulation, immune‐inflammation, and gut microbes, are discussed to extrapolate prospective homotherapy. Furthermore, the current status and challenges in various promising homotherapy for heteropathy between CVD and cancer are highlighted, especially emphasizing the use of conventional and newly developed CVD medications that may also confer benefits in cancer prevention or treatments. By bridging global trends with China‐specific insights, this review aims to advance more precise and equitable prevention and treatments, ultimately reducing the combined burden of these two interconnected diseases.

## Graphical abstract

Cardiovascular disease (CVD) and cancer are leading causes of death worldwide, with overlapping risk factors and pathophysiological mechanisms. This review explores shared pathways, including metabolic dysregulation, chronic inflammation, and gut microbiome alterations, highlighting dual‐benefit strategies such as lifestyle modifications and repurposed medications to combat both diseases. By identifying common therapeutic targets, this work aims to optimize strategies for reducing the worldwide burden of CVD and cancer.

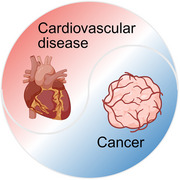

## 1 Introduction

The past decade has witnessed an epidemiological transition, marked by a decline in deaths from communicable diseases and a rise in noncommunicable diseases. Among the noncommunicable diseases, cardiovascular disease (CVD) and cancer are considered the two leading causes of mortality and morbidity worldwide.^[^ ^1^ ^]^ The global estimated number of deaths due to CVD steadily increased from ≈12.1 million in 1990 to 19.8 million in 2022.^[^ ^2^ ^]^ Notably, the increase in heart disease deaths from 2019–2020 was 10‐fold greater than the corresponding increase from 2018–2019, and this trend continues to grow. Cancer, as a primary contributor to the global disease burden after heart disease, accounts for 18% of all deaths.^[^ ^3^ ^]^ Over 35 million new cancer cases are predicted to occur by 2050 on the basis of projected population growth and aging.^[^ ^4^ ^]^ Overall, the substantial societal and macroeconomic costs of cancer are rapidly increasing globally.^[^ ^5^ ^]^

Although CVD and cancer are often considered separately and their interrelation is seldom discussed, cardiology and oncology are intricately intertwined. The morbidity of both CVD and cancer can be attributed to common risk factors such as age, diet, obesity, sedentary lifestyle, negative emotions, tobacco use and alcohol use.^[^ ^6^, ^7^ ^]^ Compared with the general population, cancer survivors (all sites), especially those diagnosed with breast, bladder, or prostate cancer, are at increased risk of developing CVD.^[^ ^8^, ^9^, ^10^, ^11^ ^]^ In the clinic, cancer treatments resulting in severe cardiovascular toxicity, such as cardiotoxicity from doxorubicin‐based chemotherapy, anti‐programmed cell death‐1 (PD‐1) immune checkpoint inhibitor (ICI) therapy^[^ ^12^ ^]^ and anti‐epidermal growth factor receptor (HER) 2 therapy‐associated cardiotoxicity, have attracted much interest.^[^ ^13^ ^]^ Biologically, several studies have shown that CVD and cancer share some common pathophysiological bases,^[^ ^14^, ^15^ ^]^ including lipid metabolism disorders, glucose metabolism disorders and inflammation. Treatments targeting these overlapping pathophysiological bases may significantly benefit the large aging population with concurrent CVD and cancer.

**Figure 1:**
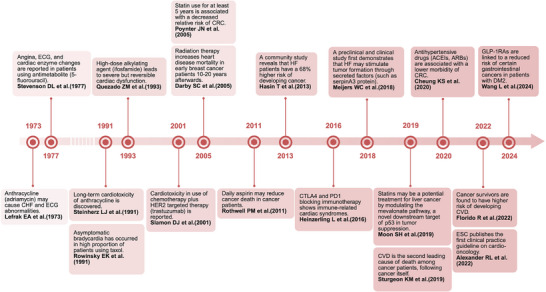
Research highlights the mechanistic and clinical intersections between CVD and cancer. Given the epidemiologically significant overlap between CVD and cancer populations with many common risk factors, research progress on the mechanistic intersections of CVD and cancer has been fuelled by a profound understanding of these two diseases with an integrated view. The past half century has witnessed clinically related research on the intersections of CVD and cancer, from revealing the cardiotoxicity of cancer treatments in the past to recently offering deeper insights into homotherapy for heteropathy in CVD and cancer. ECG, electrocardiogram; CHF, congestive heart failure; HF, heart failure; ACEI, angiotensin‐converting enzyme inhibitor; ARB, angiotensin II receptor blocker; CRC, colorectal cancer; DM2, diabetes mellitus type 2; GLP‐1RA, glucagon‐like peptide‐1 receptor agonist; ESC, European Society of Cardiology. Created with BioRender.com.

The rising clinical demand is promoting a boom in cardio‐oncology, including clinical care, scientific research, guideline formulation, cardio‐oncologist training and patient education. In a narrow sense, cardio‐oncology focuses mainly on cardiotoxicity during cancer treatment, but it is now recognized as addressing a broader correlation between CVD and cancer across all fields.^[^ ^16^ ^]^ Given the epidemiologically significant overlap between CVD and cancer with many common risk factors, the past half century has witnessed research highlighting the mechanistic and clinical intersections between CVD and cancer (**Figure** **1**). This review aims to provide a comprehensive overview of the prevalence, preventive and therapeutic strategies based on common risk factors and the shared pathophysiological bases of CVD and cancer, as well as homotherapy for heteropathy between CVD and cancer.

## 2 Search Strategy and Selection Criteria

An extensive search was conducted across MEDLINE covering the period from January 1, 2015, to May 30, 2025. Some articles of significant value were considered even if they were published more than 10 years ago. The search keywords included cardio‐oncology, cancer, “cardiovascular disease”, “risk factor”, epidemiology, “physical activity”, diet, sleep, alcohol, smoke, lifestyle, dyslipidemia, “adipose tissue”, dysglycemia, “energy metabolism”, “bile acid”, “branched‐chain amino acid”, ceramide, inflammation, efferocytosis, “clonal hematopoiesis of indeterminate potential”, “gut microbiota”, bacteria, fungi, “angiotensin‐converting enzyme inhibitors” (ACEIs), “angiotensin receptor blockers” (ARBs), digoxin, aspirin, statins, “PCSK9 inhibitors”, metformin, GLP‐1 agonists, “SGLT2 inhibitors”, paclitaxel, “anti‐PD‐1/PD‐L1 antibodies”, tipifarnib, and homotherapy. Boolean operators (e.g., AND, OR) were used to optimize the search results and ensure that the relevant literature was captured. Articles published in English were primarily selected; however, some articles in Chinese were also included because of their relevance in providing epidemiological data on Chinese populations.

## 3 The Global Problem of CVD and Cancer

CVD leads to 20.5 million deaths, accounting for approximately one‐third of all deaths worldwide, and remains a major contributor to rising healthcare costs and premature death rates.^[^ ^17^ ^]^ CVD‐related deaths are principally attributable to stroke, atrial fibrillation, cardiomyopathy, hypertensive heart disease (which ultimately progresses to heart failure), ischemic heart disease and rheumatic heart disease.^[^ ^1^ ^]^ The incidence and prevalence of CVD have increased over the past few decades, particularly among younger adults (18–50 years of age), who are more likely to develop an unhealthy cardiovascular risk profile, such as obesity.^[^ ^18^ ^]^

Cancer ranks as the second most frequent cause of death worldwide, years of life lost, and disability‐adjusted life years due to disease and injury, surpassed only by CVD.^[^ ^5^ ^]^ In 2022, there were ≈20.0 million new cancer cases and 9.7 million cancer‐related deaths worldwide.^[^ ^4^ ^]^ Despite some promising progress in early diagnosis and cancer treatment, inequities in the global cancer burden and its distribution hinder further advancements. The most common cancer, in terms of both incidence and mortality, was breast cancer in females and lung cancer in males.^[^ ^4^ ^]^

Epidemiological studies have indicated that CVD is the second most common cause of morbidity and mortality among cancer survivors following recurrent malignancy.^[^ ^19^ ^]^ In an observational study, after 3 million cancer survivors were analyzed among 28 cancer types, mainly bladder, larynx, prostate, colorectal and breast cancer, 38.0% died from cancer, and 11.3% died from CVD.^[^ ^10^ ^]^ With advancements in cancer treatment, cancer is increasingly being viewed as a chronic disease.^[^ ^20^, ^21^ ^]^ Therefore, the long‐term need for chronic disease care, such as for CVD, has become essential in cancer survivors. In a prospective study, cancer survivors (diagnosed with a first primary invasive cancer, excluding those with nonmelanoma skin cancer) had a 42% higher risk of developing CVD, particularly heart failure (HF), than individuals without cancer.^[^ ^22^ ^]^ A significant association between specific cancer subtypes and CVD has been reported among survivors of breast cancer (HR 1.58), lung cancer (HR 2.73), and hematopoietic and lymphatic cancer (HR 3.22).^[^ ^22^ ^]^ Thus, it is necessary for cancer patients to receive earlier and more aggressive cardiovascular care.

**Figure 2:**
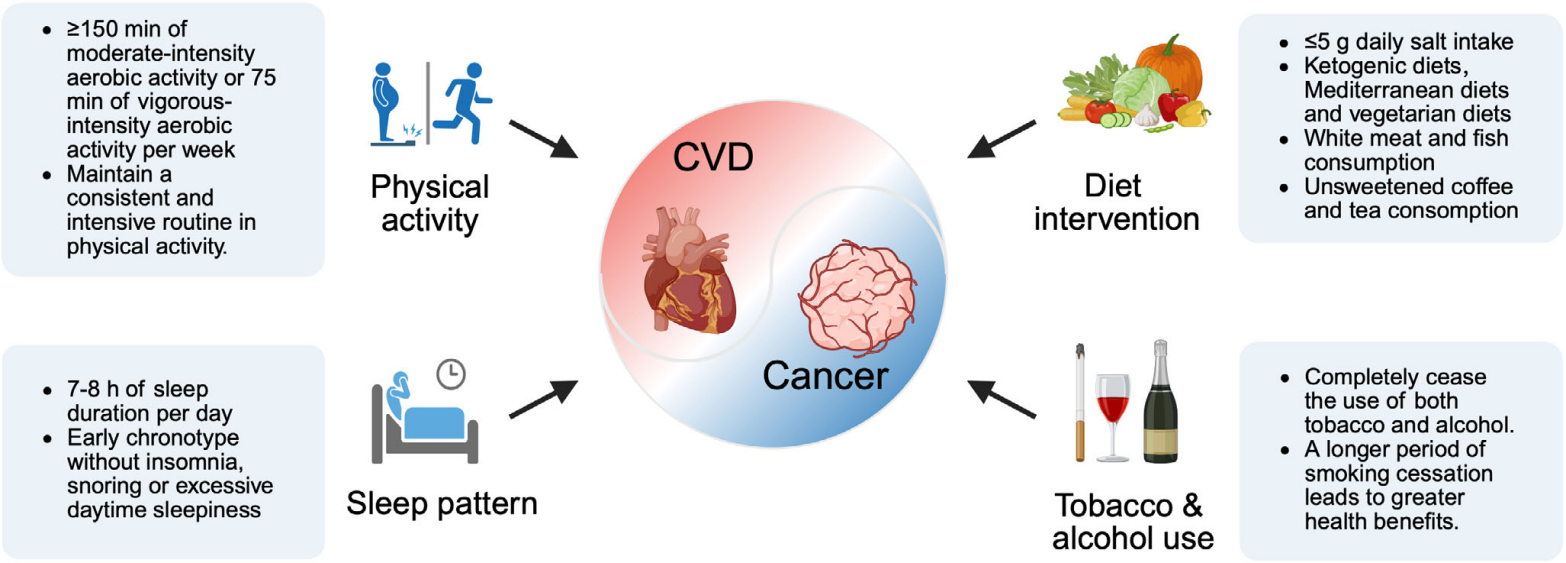
Lifestyle modifications for CVD and cancer on the basis of common risk factors. The common risk factors for CVD and cancer include age, diet, obesity, physical activity and sleep patterns, tobacco use, alcohol use, etc. Some lifestyle modifications benefit both CVD and cancer patients on the basis of common risk factors. Diet interventions include limiting daily salt intake; avoiding high‐fat diets; and adopting ketogenic, Mediterranean, or vegetarian diets. Consistent physical activity and a healthy sleep pattern are also critical. Additionally, complete cessation of tobacco and alcohol use is strongly recommended, as prolonged smoking cessation significantly enhances health outcomes. Together, these strategies form a comprehensive approach to preventing CVD and cancer. Created with BioRender.com.

Given the growing overlap between CVD and cancer populations, greater attention should be paid to their common risk factors (**Figure** **2**), as well as common prophylactic strategies and homotherapy on the basis of common pathological mechanisms.

## 4 The Demographic Disparities in CVD and Cancer Incidence in China

In China, the burdens of CVD and cancer present significant geographical disparities. Compared with that in southern China, the higher incidence of CVD in northern China may be attributed to prolonged exposure to cold climates along with long‐term consumption of high‐fat, high‐salt diets and frequent alcohol intake, which collectively increase cardiovascular risk.^[^ ^23^ ^]^ Regarding cancer, the incidence of cancer varies significantly across different provinces in China, with notable regional disparities. For example, Guangdong Province, a southern region in China, typically has a relatively high prevalence of nasopharyngeal carcinoma.^[^ ^24^ ^]^ The high incidence of nasopharyngeal carcinoma in Guangdong is attributed primarily to the region's elevated prevalence of Epstein‒Barr virus, particularly high‐risk subtypes, which thrive in warm, humid climates and have led to widespread population susceptibility due to persistent infection patterns and environmental suitability.^[^ ^25^ ^]^ Another very typical geographical difference is from mountain regions to coastal bays, which is also closely correlated with the epidemiology of CVD and cancer. Compared with mountainous regions, coastal bays generally present superior cardiovascular health indices.^[^ ^26^ ^]^ This disparity may be attributed to two primary factors. First, coastal urban centers typically benefit from enhanced economic development, which facilitates better healthcare accessibility, improved health education, and superior living conditions conducive to cardiovascular wellness. Second, high‐altitude environments present distinct physiological challenges, including chronic exposure to hypoxic and hypobaric conditions, which are frequently associated with pulmonary hypertension and HF.^[^ ^27^ ^]^ Furthermore, the prevalence of chronic mountain sickness, a pathological condition characterized by excessive erythrocytosis and cardiopulmonary dysfunction in altitude‐adapted populations, may compound cardiovascular risks in mountainous regions. There was no significant difference in the cancer disease spectrum between mountainous areas and coastal bays.^[^ ^28^ ^]^

Food ingredients and diet flavors are usually adapted to geographical conditions, while dietary differences influence CVD and cancer. China is a country where culinary flavors vary greatly among regions and is renowned for spicy, salty or sweet flavors. A discussion of food ingredients is provided in Section 5.2. Provinces with a dietary preference for spicy foods, such as Sichuan and Chongqing, tend to have higher cardiovascular health index values, possibly because capsaicin, a compound in chili peppers that has been shown to have cardiovascular protective effects.^[^ ^29^ ^]^ A prospective China Kadoorie Biobank study, which included two‐thirds of daily consumers from Hunan and Sichuan Provinces known for their spicy dietary patterns, reported that a high frequency of spicy food consumption (daily consumers) was significantly linked to a reduced risk of gastrointestinal cancer and esophageal cancer.^[^ ^30^ ^]^ This anticancer property may be attributed to capsaicin's ability to suppress *Helicobacter pylori* colonization and reduce body fat accumulation.^[^ ^31^, ^32^ ^]^ A global disease burden study on the health effects of dietary risk reported that high sodium intake is a leading dietary risk factor for deaths in China, particularly those linked to CVD and cancer.^[^ ^33^ ^]^ In China, regions with a predilection for salty flavors, such as Inner Mongolia and Ningxia, exhibit detrimental effects on CVD, particularly hypertension, which is potentially attributable to increased sodium intake. According to the China Cancer Registry Annual Report, Liaoning, Fujian, Gansu, Shandong, and Jiangsu are identified as high‐risk regions for gastric cancer,^[^ ^28^ ^]^ and the influence of prolonged consumption of salted food, including cured meats, salted fish, pickled vegetables, and preserved seafood in these areas, cannot be ruled out.

As urbanization intensifies, disparities in the distribution of CVD and cancer mortality rates between urban and rural areas become increasingly evident. Notably, CVD mortality rates are significantly higher in rural areas than in urban areas, with a discernible upward trend over the past decade. In contrast, urban CVD mortality remains relatively stable, albeit with a slight increase. Both urban and rural regions have stable mortality rates for cancer. Urban cancer mortality consistently remains slightly lower than that in rural areas. These patterns suggest differing levels of healthcare accessibility and potentially varying exposures to risk factors between urban and rural populations. Although urban residents tend to lead more sedentary lifestyles and engage in less physical activity, they typically have distinct advantages in terms of education levels and prior knowledge of CVD and cancer prevention.^[^ ^34^, ^35^, ^36^ ^]^ The superior socioeconomic status of urban residents may provide them with better access to healthcare resources and preventive measures, facilitating early diagnosis and treatment. Similarly, epidemiological surveillance data (2005–2020) revealed that age‐standardized mortality rates declined significantly for nearly all cancer types in urban areas of China, which contrasts sharply with findings in rural areas, where ≈50% of cancer types have increased mortality.^[^ ^37^ ^]^

Other lifestyle factors, such as ways of living, including herding, farming and fishing, have profound impacts on the epidemiology of CVD and cancer. Although systematic national statistics on cardiovascular and cancer incidence among Chinese herders and farmers are limited, localized studies provide preliminary insights into these regional health patterns. A study revealed a high detection rate of a high‐risk CVD population of 31.47% among Kazakh herders in the Nanshan pastoral area, Xinjiang, which markedly exceeded rates in Guangzhou (15.17%) and Beijing (20.82%).^[^ ^38^ ^]^ Cumulative effects of alcohol consumption, animal fat intake, preserved/smoked meats, and high‐fat diets were identified as the primary drivers of this elevated risk. A Taiwanese study demonstrated elevated cardiometabolic risks among agriculture and aquaculture workers, associated with low water intake, limited vegetable/fruit consumption, and prevalent betel nut chewing and smoking behaviors.^[^ ^39^ ^]^ In terms of fishing populations, China has had a limited epidemiological focus, with few domestic studies examining the characteristics of CVD. According to a previous report, fishermen in Zhoushan, Zhejiang Province, present the lowest prevalence of lower extremity atherosclerotic disease compared with other regions listed in the report.^[^ ^40^ ^]^ The harsh occupational environment, irregular diets, prolonged maritime isolation, and elevated alcohol/tobacco use among fishermen may collectively exacerbate CVD and cancer risks, significantly compromising their health.^[^ ^41^ ^]^ Although populations engaged in herding, farming, and fishing exhibit active physical activity, CVD and cancer incidence and mortality rates remain elevated compared with those of urban sedentary populations, which may be linked to lower education levels, poorer dietary habits, and higher smoking prevalence. In addition, occupational exposure to asbestos, a Group 1 carcinogen, significantly elevates health risks for workers in relevant industries. Compared with unexposed populations, prolonged asbestos exposure is strongly associated with increased incidence of lung cancer, and emerging evidence suggests potential links to CVD.^[^ ^42^, ^43^ ^]^ Notably, asbestos product manufacturers relocated from economically developed eastern China to less developed central‐western regions (e.g., Xinjiang, Gansu, Qinghai, and Sichuan) between 2010–2019.^[^ ^44^ ^]^ These regions now report disproportionately high percentages of asbestos‐related diseases, highlighting the need for increased vigilance regarding asbestos‐related CVD and cancer among workers in these regions.

## 5 Lifestyle Modifications for CVD and Cancer on the Basis of Common Risk Factors

### 5.1 Physical Activity

#### 5.1.1 Physical Activity Benefits for CVD and Cancer

Physical activity refers to any body movement generated by skeletal muscles that consumes energy. For significant health benefits regarding all‐cause mortality, adults are advised to engage in at least 150–300 min of moderate‐intensity aerobic exercise or 75–150 min of vigorous‐intensity aerobic exercise.^[^ ^45^ ^]^ A sedentary lifestyle, defined as prolonged sitting or lying down and a lack of whole‐body movement, increases the morbidity and mortality of CVD and cancer.^[^ ^46^, ^47^ ^]^ High volumes and intensities of physical activity energy expenditure lower CVD event rates.^[^ ^48^ ^]^ The risk of all‐cause mortality was reduced by 50% for coronary heart disease (CHD) patients who maintained active physical activity compared with those who remained sedentary.^[^ ^46^ ^]^ Interestingly, there was no statistically significant variation in CVD mortality between CHD patients who remained sedentary and those whose physical activity levels decreased, underscoring the importance of maintaining both routine and intense physical activity.^[^ ^46^ ^]^ A recent meta‐analysis revealed that physical activity effectively improved blood lipid levels, including elevating high‐density lipoprotein (HDL) cholesterol and reducing triglycerides and low‐density lipoprotein (LDL) cholesterol, all of which benefit cardiovascular health.^[^ ^49^ ^]^ Physical activity also affects endothelial nitric oxide synthase (eNOS)‐dependent mitochondrial biogenesis in the heart, improving glucose uptake.^[^ ^50^ ^]^ As expected, physical activity is negatively associated with cancer development. Strong evidence indicates that physical activity decreases the incidence of colon, bladder, breast, esophageal and endometrial cancers.^[^ ^51^ ^]^ At least 12 weeks of physical activity either during or after curative cancer treatment is recommended by evidence‐based international guidelines, which aim to improve health‐related quality of life and reduce cancer treatment‐related side effects.^[^ ^52^, ^53^ ^]^ A multinational randomized controlled trial involving more than 300 metastatic breast cancer patients reported that supervised physical activity (twice a week for 6 months, 1 h each time) significantly relieved patients’ fatigue and positively impacted health‐related quality of life.^[^ ^54^ ^]^ In addition, active physical activity increases carbohydrate metabolism, glycolysis and mitochondrial biogenesis in internal organs to restrict nutrient availability to tumors, thereby protecting against cancer metastasis.^[^ ^55^ ^]^

#### 5.1.2 Weight Management by Physical Activity also Benefits for CVD and Cancer Patients

Certainly, physical activity is crucial for weight management, whereas appropriate weight control to avoid obesity is beneficial for both cardiovascular health and cancer prevention. Obesity, clinically defined by the World Health Organization (WHO) as a body mass index (BMI) ≥30 kg m^−2^, and overweight, defined as a BMI ≥25 kg m^−2^ but <30 kg m^−2^, have become major public health issues owing to their steadily rising prevalence worldwide.^[^ ^56^ ^]^ Obesity is an independent risk factor for many chronic diseases, particularly CVD, type 2 diabetes mellitus (DM2), and Alzheimer's disease. It also causes dyslipidemia, insulin resistance, inflammation, and obstructive sleep apnea, which further exacerbate these diseases.^[^ ^57^, ^58^, ^59^ ^]^ For every one‐unit increase in BMI, the risk of HF increases by 5% in men and by 7% in women, demonstrating a dose‒dependent relationship.^[^ ^60^ ^]^ Currently, obesity has overtaken smoking as the most widespread risk factor for cancer.^[^ ^61^ ^]^ The International Agency for Research on Cancer (IARC) has evaluated more than 1000 epidemiological studies, revealing significant associations and positive dose‒response relationships between BMI and cancer risk, including adenocarcinoma of the kidney, colorectum, liver, gallbladder, pancreas, and esophagus.^[^ ^62^ ^]^ According to multivariable analyses, women who lost 5% or more of their body weight had a significantly lower risk of breast cancer than those who maintained weight, irrespective of BMI.^[^ ^63^ ^]^ Current weight management strategies encompass lifestyle, medication, and surgical interventions, while physical activity and diet are the two main measurements used for lifestyle modifications. Aerobic exercise can result in an average weight loss of ≈2–3 kg compared with individuals who do not engage in any physical activity or diet interventions.^[^ ^64^ ^]^ Physical activity not only increases energy expenditure but also suppresses appetite to control energy intake through inducing the production of *N*‐lactoyl‐phenylalanine (Lac‐Phe), an anorexigenic signaling metabolite.^[^ ^65^ ^]^

#### 5.1.3 Recommendation of Physical Activity for CVD and Cancer

Given the common benefits of physical activity for both CVD and cancer, it is advisable to maintain a regular physical activity routine (**Table** **1**). For primary prevention of both diseases, current clinical guidelines recommend a minimum of 150 min of moderate‐intensity aerobic activity or 75 min of vigorous‐intensity aerobic activity (including resistance exercise at least two times) per week as standard primary prevention for both CVD and cancer management.^[^ ^66^, ^67^, ^68^, ^69^ ^]^ Physical activity should also not be excluded for secondary prevention, as its benefits appear to be more pronounced among individuals with existing CVD.^[^ ^70^ ^]^ The exercise program should be optimized on the basis of the patients’ disease severity, frailty, and exercise capacity, starting with moderate‐intensity continuous training and gradually increasing the duration and intensity to meet a personalized exercise prescription.^[^ ^71^ ^]^ For patients with stable coronary artery disease (CAD), engaging in vigorous physical activity for more than 20 min per day is generally recommended.^[^ ^72^ ^]^ However, for patients with unstable CAD or severe ischemia, exercise should be suspended until their condition stabilizes. For patients with heart failure with preserved ejection fraction (HFpEF), the exercise program should begin with moderate‐intensity continuous training and gradually progress to high‐intensity interval training (HIIT) via shorter intervals and a lower target heart rate (e.g., 1‐min intervals with 75–85% maximal heart rate).^[^ ^73^ ^]^ For patients with heart failure with a reduced ejection fraction (HFrEF), the exercise program is recommended to introduce daily moderate‐intensity continuous training in a split format (e.g., three sessions of 3–5 min per day).^[^ ^71^ ^]^

**Table 1:** Physical activity recommendation for CVD and cancer.

Prevention	Diseases/conditions/symptoms	Recommended Physical Activity
Primary prevention	CVD and cancer	≥150 min/week moderate‐intensity or ≥75 min/week vigorous‐intensity, resistance exercise at least two times.^[^ ^69^ ^]^
Secondary prevention	Coronary artery disease	>20 min/day vigorous physical activity.^[^ ^72^ ^]^
	HFpEF	Start with moderate‐intensity, progress to HIIT with 1‐min intervals at 75–85% max heart rate.^[^ ^73^ ^]^ Daily moderate‐intensity training, divided into split sessions (3 sets of 3–5 min/day).^[^ ^71^ ^]^
	Cancer	150 min of moderate‐intensity aerobic exercise over 3–5 days, plus 2–3 weekly resistance training sessions targeting major muscle groups (8–10 areas), with 2 sets of 8–10 controlled repetitions per group. All exercises should start with activation and end with warm‐ups and cool‐downs.^[^ ^74^ ^]^
	Lymphedema after cancer surgery	Progressive resistance training not only unrelated to increase the incidence or severity of lymphedema but also enhances muscle strength and physical function of patients.^[^ ^76^ ^]^
	Cancer‐related fatigue	Moderate‐intensity exercise (55‐75% max heart rate, ≥30 min, ≥5 days/week) or vigorous exercise (≥20 min, ≥3 days/week), such as brisk walking or cycling.^[^ ^75^ ^]^ Engage in progressive strength training at least 3 days/week, combined with low‐intensity activities, such as yoga.^[^ ^75^ ^]^
	Cancer‐related depression, anxiety and cognitive impairment	Moderate‐intensity aerobic training performed 3 times per week for at least 12 weeks, or combined aerobic plus resistance training done twice weekly for 6‐12 weeks.^[^ ^52^, ^77^, ^78^ ^]^

HFpEF, heart failure with preserved ejection fraction; HIIT, high‐intensity interval training.

For cancer patients, exercise programs are needed as a routine part of care.^[^ ^53^ ^]^ According to the clinical guidelines in Canada, cancer patients are recommended to perform not only 150 min of moderate‐intensity aerobic exercise spread over 3–5 days but also resistance training targeting major muscle groups (8–10 distinct areas) 2–3 times per week, with each muscle group completing two sets of 8–10 controlled movements. All exercise routines must begin with preparatory activation exercises and conclude with warm‐ups and cool‐downs.^[^ ^74^ ^]^ Even during active anticancer therapy, endurance and strength exercises are still recommended for patients, given their ability to enhance the curative effects of anticancer therapy and reduce adverse effects.^[^ ^53^ ^]^ For example, to relieve cancer‐related fatigue, which frequently occurs in anticancer therapy, patients are encouraged to perform moderate‐intensity exercise (55–75% max heart rate, ≥30 min, ≥5 days per week) or vigorous exercise (≥20 min, ≥3 days per week), such as brisk walking or cycling. Progressive strength training for at least 3 days per week combined with low‐intensity activities (e.g., yoga) can also alleviate cancer‐related fatigue during treatment and improve survival after treatment.^[^ ^75^ ^]^ Certain exercises can aid in recovery after cancer surgery. In contrast to the previous belief that breast cancer patients should restrict exercise in the affected arm, breast cancer patients are now advised not to restrict strenuous exercise in the affected arm, which is based on recent evidence that progressive resistance training is not only unrelated to an increased incidence or severity of lymphedema but also enhances the muscle strength and physical function of patients.^[^ ^76^ ^]^ Interestingly, exercise interventions also have positive effects on reducing depression, anxiety severity and cancer‐related cognitive impairment in cancer patients,^[^ ^52^, ^77^, ^78^ ^]^ which are usually set as moderate‐intensity aerobic training or combined aerobic plus resistance training^[^ ^52^, ^79^, ^80^ ^]^ A related study in which breast cancer patients participated in a 12‐week exercise program suggested aerobic exercise (50–60% max heart rate, 50 min, 3 days per week) and resistance training (≥2 days per week, 60 min). The resistance training consisted of a 10‐min warm‐up, followed by a 40‐min leg and hip workout using an elastic band and ball, and then a 10‐min cool‐down. The results revealed that, compared with those in the control group, the depression levels of the participants in the exercise group were lower.^[^ ^81^ ^]^

There are still many ongoing clinical trials investigating the impact of exercise on various cancers and their therapies. Nevertheless, exercise prescriptions and recommendations for CVD and cancer patients in clinical practice are still not precisely individualized and do not consider dynamic disease progression, concurrent comorbidities, or treatments with different exercise tolerances. Medical providers still need training to provide high‐quality exercise counseling. With the advancement of artificial intelligence technology, we look forward to large models that, on the basis of more data from clinical research or the real world, can support precise individualized exercise recommendations tailored to CVD and cancer patients.

### 5.2 Diet Modification

“A closed mouth catches no flies.” Ancient wisdom and modern scientific research indicate that a healthy diet has crucial effects on disease prevention and treatment, especially for chronic diseases. CVD and cancer are among the most common chronic diseases. Over the past few decades, epidemiological studies have linked various dietary patterns to CVD and cancer. For example, certain food sources, such as nitrosamines and aflatoxins, are well‐established carcinogens, and excessive dietary sodium is a major trigger of hypertension and CVD.^[^ ^33^ ^]^ On the basis of the current reality that cancer patients have a significant burden of CVD, understanding the overlap of dietary patterns and components that significantly influence disease risk, prevention, progression and treatment in both CVD and cancer is crucial for providing better dietary interventions for patients who are at risk of both conditions.^[^ ^6^, ^16^ ^]^ Here, we introduce four common dietary interventions that influence CVD and cancer, including salt intake, high‐fat diets, vegetarian diets and ketogenic diets.

#### 5.2.1 Salt Intake

Modulating salt intake is a potent intervention for the prevention of both CVD and cancer. The recommended daily salt intake for healthy adults is less than 5 g d^−1^, but the majority of the population exceeds this standard.^[^ ^82^, ^83^ ^]^ A J‐shaped relationship was observed between sodium intake and CVD incidence, showing minimal risk at 3–5 g of sodium (≈7–12 g of salt) intake per day.^[^ ^84^ ^]^ Several blood pressure guidelines recommend low sodium intake for the entire population, suggesting a daily limit of less than 2.3 g, which is approximately equivalent to 5 g of salt.^[^ ^82^ ^]^ Reducing dietary salt by 3 g d^−1^ is projected to reduce the annual number of new CVD cases and save healthcare costs across all population segments.^[^ ^85^ ^]^ The association between elevated CVD risk and high sodium consumption is believed to result from a reduction in the effectiveness of the renin‐angiotensin‐aldosterone system, which leads to a subsequent increase in cardiac output.^[^ ^86^ ^]^ Gastric cancer patients are also victims of high‐salt diets.^[^ ^87^, ^88^, ^89^ ^]^ High salt intake may promote the colonization of *Helicobacter pylori* in the stomach and directly damage the gastric mucosa, which increases the possibility of endogenous mutations. Similarly, colorectal cancer (CRC) survivors are also recommended to consume no more than 6 g of salt per day.^[^ ^90^ ^]^ Nevertheless, extensive research on the correlation between the amount of salt intake and patient survival is lacking in most cancer types.

#### 5.2.2 High‐Fat Diet

High‐fat diets, which contain high levels of saturated fats and cholesterol but low levels of fiber, minerals and vitamins, are widely acknowledged for their detrimental effects on CVD, particularly atherosclerosis. In addition, high‐fat diets are also associated with an increased risk of cancer development.^[^ ^91^ ^]^ High‐fat diets contribute to immunosuppressive tumor microenvironments (TMEs). In a CRC mouse model, a high‐fat diet accelerated tumor growth by reducing the quantity and functionality of intratumoral CD8^+^ T cells.^[^ ^92^ ^]^ High‐fat diets can also promote cancer growth and metastasis by altering the composition of the gut microbiota.^[^ ^93^ ^]^ *Lactobacillus* was reported to decrease in the stomachs of mice fed a high‐fat diet.^[^ ^94^ ^]^ A recent study revealed that *Lactobacillus johnsonii* enhances the efficacy of ICI therapy via CD8^+^ T cells across various cancer types.^[^ ^95^ ^]^ *Lactobacillus* has cholesterol‐lowering properties and shows promise as a next‐generation probiotic for preventing or treating CVD.^[^ ^96^ ^]^ In a mouse model of obesity induced by a high‐fat, high‐fructose diet, supplementation with *Lactobacillus rhamnosus GG* ameliorated myocardial function, highlighting its cardioprotective effects.^[^ ^97^ ^]^ For cardiovascular health, linoleic acid‐rich oils are preferred.^[^ ^83^ ^]^ However, a recent study indicated that safflower oil (rich in linoleic acid) induced T‐cell mitochondrial dysfunction and promoted mammary tumor growth.^[^ ^98^ ^]^ To promote cardiovascular health, limiting total fat intake to 20–35% of daily calories, prioritizing liquid plant oils (such as olive, corn, and safflower) over animal fats (butter) and tropical oils (coconut), and restricting saturated fat to <10% while avoiding trans fats are recommended.^[^ ^99^ ^]^ Limiting high‐fat foods and trans fats may lower the risk of certain cancers, which aligns with the recommendations for cardiovascular health.^[^ ^100^ ^]^

#### 5.2.3 Vegetarian Diet

The vegetarian diet is gaining popularity not only for its environmentally friendly impact but also for its potential health advantages. Vegetarians were reported to have a lower risk of CVD, especially ischemic heart disease, than meat eaters, according to prospective analyses.^[^ ^101^, ^102^ ^]^ Similarly, a vegetarian diet has been linked to reduced cancer risk. In the EPIC‐Oxford (European Prospective Investigation into Cancer and Nutrition Oxford cohort) study, vegetarians presented a 10% reduction in the risk of all‐site cancers compared with meat eaters, whereas vegans presented an 18% reduction.^[^ ^103^ ^]^ This reduction may be attributed to the lower circulating levels of insulin‐like growth factor‐I (IGF‐1) observed among vegans. Elevated IGF‐1 levels are associated with an increased risk of colorectal, breast, and prostate cancers, suggesting that the lower IGF‐1 levels in vegans could be a contributing factor to their reduced cancer risk.^[^ ^101^, ^104^ ^]^ In dietary guidelines for both cancer and CVD, there is a strong emphasis on the consumption of a diverse array of vegetables and fruits in ample quantities^[^ ^99^, ^100^, ^105^, ^106^ ^]^ (**Table** **2** **)**. Citrus fruits, which have a low glycemic index, are highly recommended for their potential to decrease CVD incidence and lung cancer risk.^[^ ^107^, ^108^ ^]^ Numerous studies have highlighted the cardioprotective benefits of specific vegetable groups, including allium (e.g., garlic and onion), carrot, cruciferous vegetable (e.g., broccoli and kale), and green leafy vegetable groups.^[^ ^108^ ^]^ Current guidelines recommend a daily intake of at least 300–400 g of vegetables or fruits to mitigate CVD and cancer risks, respectively.

**Table 2:** Dietary recommendation for CVD and cancer.

Food item	CVD	Cancer
Salt	For hypertension prevention: ≤ 5 g per day.^[^ ^82^ ^]^ For the lowest risk of CVD incidence and mortality: 7‐12 g per day.^[^ ^84^ ^]^	For colorectal cancer survivors: no more than 6 g per day.^[^ ^90^ ^]^ No evidence for many cancers.
Dietary fats	Oils (unsaturated fatty acids sources): 45 g per day.^[^ ^83^ ^]^ Restrict saturated fat <10% daily calories and avoid trans fats.^[^ ^99^ ^]^ Recommend linoleic acid rich oils.^[^ ^83^ ^]^	Safflower oil (rich in linoleic acid) induces T‐cell mitochondrial dysfunction and promotes mammary tumor growth.^[^ ^98^ ^]^
Cereals	High GI refined cereals (white rice and white bread): limit to no more than 2 servings per week (the less the better). Low GI refined cereals (pasta, parboiled rice, and corn tortilla): 1‐2 servings per day. Whole grains (bread, rice, oat, and barley): 2 servings per day It is recommended to replace high GI refined cereals with both low GI and whole‐grain cereal foods.^[^ ^83^ ^]^	A daily intake of 50‐90 g of whole grains is associated with a 9‐20% lower cancer mortality risk.^[^ ^130^ ^]^ 90 g per day consumption of refined grains was associated with a 6% lower risk of total cancer.^[^ ^131^ ^]^
Vegetables and fruits	At least 400 g each of vegetables and fruits per day.^[^ ^83^ ^]^ Legumes should be consumed in larger quantities.^[^ ^83^ ^]^ Recommended fruit sources: citrus, 100% fruit juice, and pommes.^[^ ^108^ ^]^ Recommended vegetable sources: allium, carrots, cruciferous, and green leafy.^[^ ^108^ ^]^	At least 300 g each of vegetables and fruits per day.^[^ ^105^ ^]^ Consuming 3‐5 or more servings of dried fruits may reduce pancreatic cancer mortality risk, as well as the incidence of stomach and nasopharyngeal cancer.^[^ ^106^ ^]^ Consuming 60 g of citrus per day may decrease lung cancer risk, but intakes over 80 g per day do not show an obvious does‐response association.^[^ ^107^ ^]^
Meat	White meat (chicken, turkey and rabbit) is preferred to red meat (beef, pork and lamb).^[^ ^83^ ^]^ White meat: moderate amounts (up to 300 g per week).^[^ ^83^ ^]^ Displace processed meat (bacon, sausages and salami).^[^ ^83^ ^]^	Red meat and processed meat are not recommended.^[^ ^124^ ^]^ White meat may decrease the risk of certain cancers and related mortality.^[^ ^124^ ^]^
Fish	300‐600 g per week as a means to contribute to atherosclerosis prevention.^[^ ^83^ ^]^ Preferably oily types^[^ ^125^ ^]^	Omega‐3 rich in the fish oil decrease the protumor macrophages in breast cancer mouse models.^[^ ^126^ ^]^ Pescatarian diet showed a lower risk of all cancers compared with meat‐eaters.^[^ ^127^ ^]^
Nut	30 g per day^[^ ^83^ ^]^ Walnuts is relatively more recommended.^[^ ^134^ ^]^	56‐60 g per week^[^ ^135^ ^]^ Brazil nuts are excellent source of selenium, which decrease colorectal cancer risk.^[^ ^136^ ^]^ Tree nut is more recommended than peanut.^[^ ^137^ ^]^
Coffee and tea	Green tea is more recommended for its high catechin content to prevent atherosclerosis.^[^ ^131^ ^]^ Daily consumption of 2.5‐3.5 cups of unsweetened coffee is most strongly associated with a reduced risk of CVD mortality.^[^ ^128^ ^]^	Tea consumption has protective effects on certain types of cancer, especially oral cancer.^[^ ^129^ ^]^ Daily consumption of 2.5‐3.5 cups of unsweetened coffee is most strongly associated with a reduced risk of cancer mortality.^[^ ^128^ ^]^

CVD, cardiovascular disease; GI, glycemic index.

#### 5.2.4 Ketogenic Diet

The ketogenic diet, a low‐carbohydrate and high‐fat diet with adequate protein to place the body in a state of nutritional ketosis, is well tolerated by patients with multiple types of cancer.^[^ ^109^ ^]^ Minimal carbohydrate consumption induces ketone production and reduces glucose levels, which suppresses cancer development and progression. A ketogenic diet has significant advantages in protecting healthy cells and reducing tumor growth when combined with traditional chemotherapy and/or radiotherapy.^[^ ^110^, ^111^ ^]^ The median survival time of a pancreatic cancer mouse model on a ketogenic diet with gemcitabine was 42% longer than that of mice on a control diet, suggesting that a ketogenic diet could be a beneficial adjunct to chemotherapy or a second‐line treatment for pancreatic cancer.^[^ ^112^ ^]^ A case series of 37 patients with various cancers (including lung, breast, colorectal and pancreatic cancer) evaluated as stage IV supported that the ketogenic diet significantly improved survival, with a median overall survival of 32.2 months, a 3‐year survival rate of 44.5%, and potential synergistic effects with chemotherapy.^[^ ^113^ ^]^ It also improves the efficacy of anti‐cytotoxic T lymphocyte antigen‐4 (CTLA‐4) immunotherapy by activating the adenosine monophosphate–activated protein kinase (AMPK) pathway to decrease PD‐1 ligand 1 (PD‐L1) protein levels.^[^ ^114^ ^]^ Ketosis has anti‐inflammatory effects that improve the response to cancer treatment and prevention.^[^ ^109^ ^]^ The principal anti‐inflammatory mechanism of β‐hydroxybutyrate involves its inhibitory action on the NLRP3 inflammasome, a central regulator of proinflammatory cytokines.^[^ ^115^ ^]^ Activation of the NLRP3 inflammasome plays a significant role in cardiac pathology, contributing to the deterioration of myocardial function and the progression of CVD.^[^ ^116^, ^117^ ^]^ However, a ketogenic diet has conflicting effects on breast cancer progression, as it suppresses primary tumor growth but enhances the metastatic capacity of cancer cells.^[^ ^118^ ^]^ Interestingly, mice fed a ketogenic diet accumulate senescent cells via the activation of p53 and p21 in multiple organs and normal tissues, especially in the heart and kidney.^[^ ^119^, ^120^ ^]^ Evidence indicates that low‐carbohydrate diets elicit proinflammatory responses in mice, potentially increasing the risk of cardiac fibrosis.^[^ ^121^, ^122^ ^]^ However, Goldberg et al. emphasized that the duration of a ketogenic diet is the key determinant in determining whether it exerts proinflammatory or anti‐inflammatory effects.^[^ ^123^ ^]^ A short‐term ketogenic diet improved murine metabolism by activating tissue‐resident γδ T cells, whereas prolonged adherence to the ketogenic diet led to systemic inflammation, obesity, and glucose intolerance by depleting γδ T cells.^[^ ^123^ ^]^ While several studies suggest that a ketogenic diet may improve CVD and cancer risk factors, long‐term efficacy remains debated owing to limited preclinical and clinical trials, unclear mechanisms for comorbid conditions, and poor adherence for patients, which weakens the strength of clinical recommendations.

#### 5.2.5 Food Recommendations for CVD and Cancer

Given the diverse dietary patterns and their varying impacts on health, it is essential to delve into the specific impacts of individual ingredients, particularly those associated with meat and fish consumption. Meat can be categorized into red (pork, beef and lamb) or white (rabbit, turkey and chicken) types according to its nutritional profile (fat, cholesterol and iron). Research indicates that white meat is associated with lower risks of CVD and certain cancers (e.g., colorectal and liver cancer) compared to red meat.^[^ ^124^ ^]^ Additionally, meat can be consumed in either fresh or processed forms. However, processed meats, which often contain added salt and various chemicals, are generally discouraged because of their negative associations with both CVD and cancer. With respect to fish consumption, multiple meta‐analyses have demonstrated that moderate intake is associated with a decreased risk of CHD incidence and mortality, with fatty fish rich in omega‐3 fatty acids providing particularly strong cardioprotective effects.^[^ ^125^ ^]^ Omega‐3 fatty acids derived from fish oil have also been shown to reduce the number of protumor macrophages in mouse models of breast cancer.^[^ ^126^ ^]^ Compared with meat eaters, individuals who adhere to a pescatarian diet have a lower overall risk of developing various cancers.^[^ ^127^ ^]^

Diet is an integral aspect of daily life. In addition to the modification of main meals, research has shown that coffee consumption offers significant health benefits. Compared with nonconsumer consumption, a daily intake of 2.5 to 3.5 cups of unsweetened coffee could lower all‐cause mortality by 29%, cancer mortality by 31% and CVD mortality by 34%.^[^ ^128^ ^]^ Tea consumption, especially the consumption of green tea, is beneficial for treating atherosclerosis and certain cancers, such as oral cancer.^[^ ^129^ ^]^ This benefit is attributed to the high catechin content in green tea.^[^ ^130^, ^131^ ^]^ However, popular milk teas in China should be distinguished carefully because of their high sugar and fat contents, along with various additives, which may have harmful effects on CVD and cancer. Moreover, special ingredients such as nuts, cereals, and sugar‐sweetened beverages are also associated with CVD incidence and/or mortality^[^ ^132^, ^133^, ^134^, ^135^, ^136^, ^137^ ^]^(Table 2). Collectively, diet plays a crucial role in the development and progression of diseases, particularly CVD and cancer, where common diet modifications can improve outcomes for both. By carefully selecting an appropriate diet, individuals can potentially enhance cardiovascular health and reduce the risk of cancer.

### 5.3 Sleep Pattern Changes

Sleep, as a substantial part of our life, plays an important role in metabolic processes and overall health. Sleep patterns, involving quality, duration and timing, have recently been proven to be correlated with CVD, cancer and all‐cause mortality, garnering extensive attention. The European Heart Journal defines a healthy sleep pattern as 7–8 h of sleep per day, an early chronotype without insomnia, snoring or excessive daytime sleepiness.^[^ ^138^ ^]^ Individuals with a healthy sleep pattern can avert more than 10% of CVD events, whereas those with recurrent insomnia, daytime sleepiness or snoring are at the highest risk of CVD.^[^ ^138^ ^]^ Inadequate sleep duration, frequent insomnia symptoms, a late chronotype, and excessive daytime sleepiness, which have become common phenomena in modern society, can impair health and reduce cancer survival.^[^ ^139^ ^]^ Sleep duration demonstrated a U‐shaped dose‒response pattern with respect to mortality, with the minimum hazard for CVD and cancer observed to be lowest among those who slept 7–8 h per day.^[^ ^140^, ^141^ ^]^ Compared with individuals who sleep 7–8 h per day, individuals who sleep less than 7 h per day are 69% more likely to develop cancer.^[^ ^142^ ^]^ Conversely, prolonged sleep duration (exceeding 9 h per day) resulted in a higher incidence of total stroke.^[^ ^143^, ^144^, ^145^ ^]^

Shift workers, who often work early morning, evening or night shifts, frequently disrupt their daily routines, as they attempt to maintain a daytime social life on their days off. This disruption, along with nighttime light exposure, can disturb the body's natural circadian rhythm, decreasing melatonin levels and deregulating circadian genes involved in cancer‐associated signaling.^[^ ^146^ ^]^ Melatonin suppression further contributes to immune system deficiencies that inhibit the function of natural killer cells and alter the balance of T helper (Th) 1 to Th2 cytokines.^[^ ^147^, ^148^, ^149^ ^]^ Epidemiological studies have confirmed that long‐term shift work is correlated with a greater risk of CVD and cancer.^[^ ^150^, ^151^ ^]^ A late chronotype disrupts circadian clocks in cardiac, endothelial and smooth muscle cells, leading to atherosclerosis, hypertension and vascular aging.^[^ ^152^, ^153^ ^]^ Unhealthy sleep patterns are associated with elevated serum inflammatory markers and poor glycemic control, particularly in those with a late chronotype, which increases mortality from both CVD and cancer.^[^ ^141^, ^154^, ^155^ ^]^ Thus, maintaining healthy sleep patterns through adequate sleep duration (7–8 h), early chronotype, high‐quality sleep and regular sleep schedules could improve outcomes for CVD and cancer patients (**Table** **3** **)**.

**Table 3:** Recommendation of sleep patterns for CVD and cancer patients.

	CVD and cancer
Recommended	7‐8 h of sleep duration per day^[^ ^140^, ^141^ ^]^ Early chronotype^[^ ^138^ ^]^ Regular sleep schedules^[^ ^146^ ^]^
Not recommended	Insomnia^[^ ^138^ ^]^ Snoring^[^ ^138^ ^]^ Excessive daytime sleepiness^[^ ^139^ ^]^ Late chronotype^[^ ^141^, ^154^, ^155^ ^]^ Prolonged sleeping duration (exceeding 9 h per day)^[^ ^143^, ^144^, ^145^ ^]^

### 5.4 Reduction in and Cessation of Tobacco and Alcohol Use

Tobacco and alcohol are integrated into daily life worldwide, although both are prone to abuse. Tobacco use is a well‐known deleterious risk factor for CVD and cancer.^[^ ^156^, ^157^, ^158^, ^159^ ^]^ Over 84% of women and 90% of men with lung cancer have a history of smoking.^[^ ^160^ ^]^ Tobacco‐induced carcinogens can be absorbed into the bloodstream and eventually excreted in the urine. Prolonged exposure to carcinogens in the urothelium of the bladder increases the risk of cellular mutations and bladder cancer.^[^ ^161^ ^]^ Approximately 10% of all CVD cases worldwide can be attributed to smoking.^[^ ^162^ ^]^ The Atherosclerosis Risk in Communities (ARIC) study analyzing more than 13 thousand participants without three major atherosclerotic diseases (peripheral artery disease, stroke and coronary heart disease) at baseline indicated that, after a median follow‐up of 26 years, individuals who smoked more than 40 pack‐years (packs per year of smoking in years) had a 4‐fold elevated risk of peripheral artery disease, 2.1‐fold for CHD and 1.8‐fold for stroke.^[^ ^163^ ^]^ Although former smokers still have a higher incidence of CVD than nonsmokers do, the good news is that they exhibit markedly lower lung cancer mortality and incident CVD rates than current smokers do.^[^ ^164^, ^165^ ^]^ A longer period of smoking cessation is associated with a lower risk of developing atherosclerotic diseases.^[^ ^163^ ^]^ Former smokers with CVD who quit within the past 25 years had a greater risk of developing lung cancer than those without CVD did, but those who quit for over 25 years did not have an increased risk of lung cancer, similar to the risk profile of nonsmokers.^[^ ^166^ ^]^ Smoking cessation (at least 3 weeks prior) also reduces morbidity and mortality after lung cancer surgery.^[^ ^167^ ^]^ All the results underscore the substantial health advantages of smoking cessation.

Alcohol use also has a significant effect on health. In 1988, the IARC categorized alcohol as a Group 1 carcinogen, the highest level of classification.^[^ ^168^ ^]^ Bagnardi et al. associated every category of alcohol consumption, from light (≤12.5 g per day) to heavy (>50 g per day), with a dose‐dependent increase in breast, oropharyngeal and esophageal squamous cell carcinomas, whereas moderate (≤50 g per day) and heavy drinking were associated with an increased incidence of liver, stomach, and gallbladder cancer.^[^ ^169^ ^]^ Interestingly, individuals who consume light alcohol even have lower risks of kidney and thyroid cancers than nondrinkers or occasional drinkers do.^[^ ^169^ ^]^ Several meta‐analyses of observational studies have demonstrated that alcohol consumption may have cardioprotective effects on CVD, often depicted as a J‐shaped curve.^[^ ^170^, ^171^, ^172^ ^]^ This curve decreases sharply and then increases gradually with increasing alcohol consumption. An average intake of <30 g d^−1^ without episodes of heavy drinking is associated with the lowest risk of ischemic heart disease.^[^ ^173^ ^]^ However, a recent large‐scale observational study has challenged the notion of the cardioprotective benefits of alcohol, suggesting that the observed reduction in CVD risk may actually be attributed to other lifestyle factors associated with light‐to‐moderate drinking habits.^[^ ^174^ ^]^ Wood et al. reported that increased alcohol consumption followed a dose‒response curve, instead of a J‐shaped curve, with a greater risk of all stroke subtypes and HF.^[^ ^175^ ^]^

Since alcohol and tobacco use often cooccur and the synergistic effects of multiple factors are challenging to separate in research, the results can lead to biases and controversies. Therefore, advocating for the complete cessation of both tobacco and alcohol use is generally more prudent, as this certainly reduces the risk of CVD and cancer.

## 6 Prospective Homotherapy Based on Intersectional Mechanisms Between CVD and Cancer

### 6.1 Metabolic Regulation Enables Metabolism‐Targeted Homotherapy for CVD and Cancer

**Figure 3:**
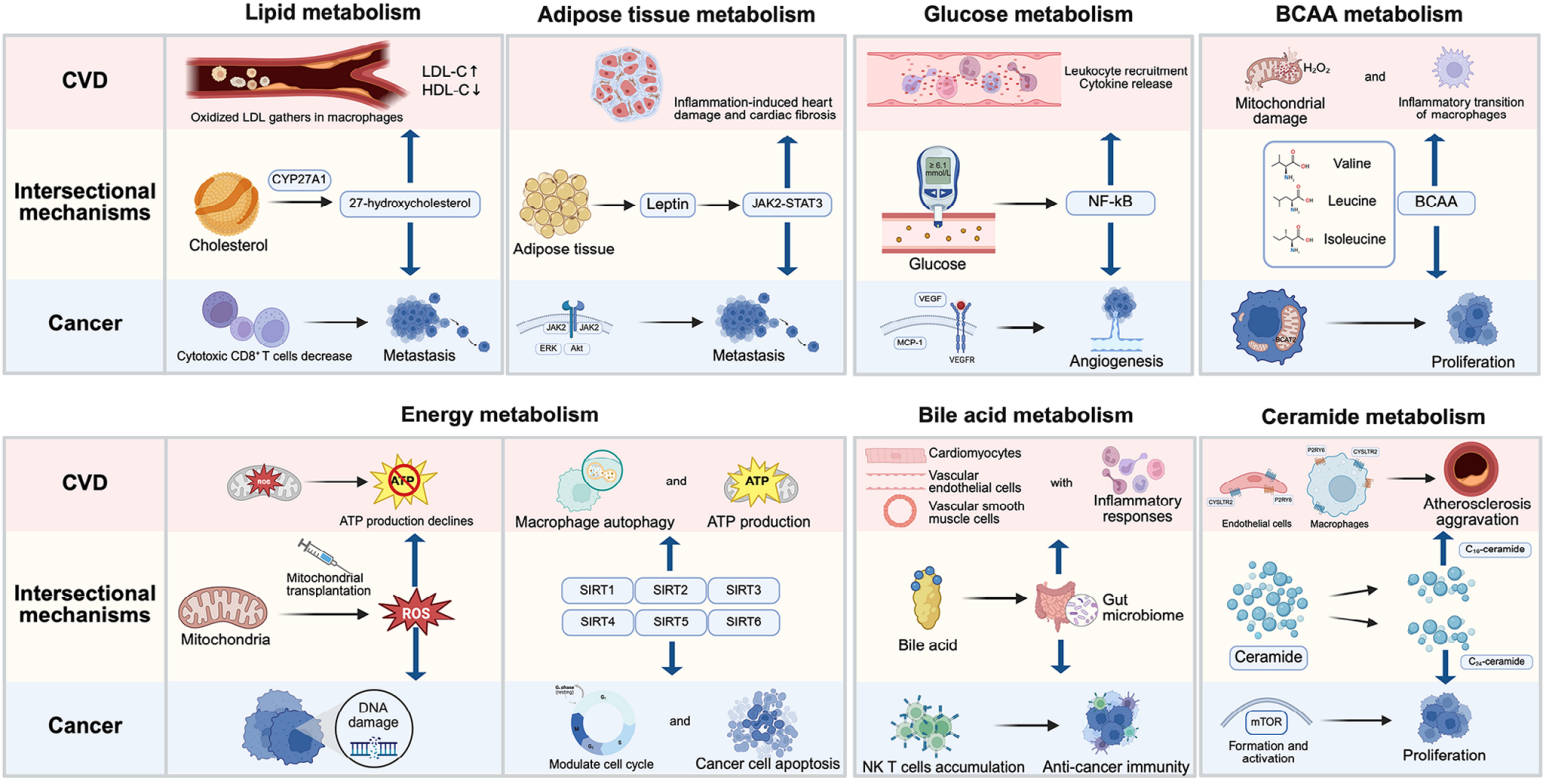
Metabolic regulation enables metabolism‐targeted homotherapy for CVD and cancer. This figure illustrates the interplay of dyslipidemia, adipose tissue metabolism, hyperglycemia, energy metabolism, bile acid metabolism, ceramide metabolism, and BCAA metabolism in CVD and cancer. The shared metabolic abnormalities suggest potential targets for homotherapy to manage both diseases. LDL‐C, low‐density lipoprotein cholesterol; HDL‐C, high‐density lipoprotein cholesterol; NK, natural killer; ROS, reactive oxygen species. Created with BioRender.com.

Metabolic regulation is a common feature of both CVD and cancer. In recent years, research has increasingly recognized the crucial role of metabolism, which has become a hot topic in the scientific community. Understanding the metabolic interplay between CVD and cancer reveals novel insights into the biological mechanisms that explain their coexistence and mutual impact, which is critical for advancing prevention strategies and therapies for both diseases (**Table** **4**). We discuss shared abnormalities in various metabolisms, as well as their potential treatments relevant to CVD and cancer below (**Figure** **3**).

**Table 4:** The mechanistic intersection of CVD and cancer in terms of metabolic remodeling, immune‐inflammation and gut microbes.

Target	Intersectional mechanisms	Impact on CVD	Impact on cancer
The mechanistic intersection of CVD and cancer in metabolic remodeling			
Blood Lipid	Cholesterol	LDL cholesterol↓ → incidence of CVD↑ HDL cholesterol↑ → incidence of CVD and all‐cause mortality reflects a U‐shaped relationship	Circulating plasma cholesterol levels↑ →mortality risk in breast, ovarian, pancreatic and prostate cancers↑^[^ ^180^ ^]^
	Oxidized LDL	Pro‐inflammatory responses and excessive macrophage apoptosis → initiate atherosclerosis lesions^[^ ^177^ ^]^	Proliferation, migration and invasion of breast, prostate, ovarian, thyroid and pancreatic cancer↑^[^ ^180^ ^]^ Breast cancer cell stemness↑^[^ ^180^ ^]^
	27‐hydroxycholesterol	Hypercholesterolemia‐driven endothelial activation, monocyte recruitment and foam cell formation → atherosclerosis^[^ ^182^ ^]^	Breast cancer and melanoma cell capacity for tumorigenesis and metastasis↑^[^ ^181^ ^]^
Adipose	Leptin	Maintaining physiological leptin is vital for normal cardiovascular function.^[^ ^186^ ^]^	Leptin↑→ proliferation, migration and invasion of endometrial cancer, breast cancer, and melanoma↑^[^ ^184^ ^]^
Glucose	NF‐κB	Hyperglycemia → NF‐κB↑ → endothelial inflammation^[^ ^197^ ^]^	NF‐κBp65↑→ aerobic glycolysis↑→lactate accumulation↑→ PI3K/Akt↑→ HBV‐related hepatocellular carcinoma cell proliferation↑^[^ ^199^ ^]^ NF‐κB↑→angiogenesis^[^ ^200^ ^]^
Energy	ROS	ROS↑ → mitochondrial ATP production in heart failure↓^[^ ^206^ ^]^	Cancer cell has higher ROS level than normal cell.^[^ ^204^ ^]^ ROS→ tumorigenesis and progression in breast, prostate, and ovarian cancer
	SIRT1	SIRT1 exerts protective effects on myocardial ischemia‒reperfusion injury.^[^ ^208^ ^]^ SIRT1↑ → cardiac dysfunction^[^ ^208^ ^]^	SIRT1‐FOXO3‐BNIP3↓ → mitochondrial dysfunction and cell death in hepatocellular carcinoma^[^ ^209^ ^]^
	SIRT3	Mitochondrial function and ATP production↑^[^ ^211^ ^]^ ROS production↓	Hepatocellular carcinoma development↓^[^ ^212^ ^]^
	SIRT4	NF‐κB/IκB/CXCL2/3 pathway↓ → vascular inflammation↓^[^ ^214^ ^]^	SIRT4 disrupt glutamine metabolism to suppress hepatocellular carcinoma.^[^ ^213^ ^]^
	SIRT6	Macrophage autophagy → endothelial inflammation and foam cell formation↓ → atherosclerosis progression↓^[^ ^215^ ^]^	Notch signaling pathway↑ → prostate cancer progression^[^ ^217^ ^]^ Aerobic glycolysis↓ → melanoma cells growth↓^[^ ^216^ ^]^
	PINK1‐Parkin	Mitophagy↑ → the engraftment ability of endothelial cells↑ → beneficial for ischemia and myocardial infarction^[^ ^218^ ^]^	Mitochondrial transplantation treatment →PINK1 and Parkin↑ → tumorigenesis in breast cancer↓^[^ ^219^ ^]^
Bile acids	Gut microbiota	Intestinal dysbiosis→ cardiotoxic bile acids level↑ → CVD^[^ ^229^ ^]^	Microbiota‐dependent bile acids→ carcinogenesis in gastrointestinal cancer^[^ ^224^ ^]^
	Immunological homeostasis	DCA → NLRP3↑ → interleukin 1β↑ → atherosclerosis^[^ ^235^ ^]^ LCA → TGR5↑ → NLRP3↓→ cytokine↓→ protecting against on atherosclerotic plaque^[^ ^235^ ^]^	Primary bile acids→CXCL16↑ → hepatic NKT cells↑^[^ ^233^ ^]^ Secondary bile acids→CXCL16↓ → hepatic NKT cells↓^[^ ^233^ ^]^ Primary bile acids → oxidative stress and T‐cell death in liver cancer model^[^ ^234^ ^]^ Secondary bile acids → promote T‐cell dysfunction in liver cancer model^[^ ^234^ ^]^
Ceramide	Apoptotic modulation	ASCVD risk stratification^[^ ^238^ ^]^ CYSLTR2 and P2RY6 receptors activation → atherosclerosis^[^ ^239^ ^]^ Cardiac hypertrophy and dysfunction^[^ ^240^ ^]^	Apoptosis in head and neck squamous cell carcinoma, breast cancer, non‐small cell lung cancer, colorectal cancer, brain cancer, ovarian cancer, and melanoma^[^ ^241^ ^]^ C24‐ceramide↑→ proliferation and migration of gallbladder cancer↑^[^ ^242^ ^]^ Ceramide↑→ immunosuppressive tumor microenvironment →breast cancer progression↑^[^ ^243^ ^]^
BCAA	Energy production and signaling pathway modulation	Atherosclerosis progression,^[^ ^248^ ^]^ thrombosis formation^[^ ^249^ ^]^ and heart failure exacerbation^[^ ^250^ ^]^↑	The risk of pancreatic cancer^[^ ^246^ ^]^ and lung cancer^[^ ^247^ ^]^↑
The mechanistic intersection of CVD and cancer in immune‐inflammation			
Macrophages	Efferocytosis	The clearance of apoptotic cells↑→ Atherosclerosis↓^[^ ^258^ ^]^	Gastric cancer peritoneal metastases↓^[^ ^262^ ^]^ M1‐like macrophage‐mediated local/remote antitumor effects in small cell lung cancer↑^[^ ^263^ ^]^
Interleukins	IL‐1	CANTOS: canakinumab (IL‐1 antibody) → recurrent cardiovascular events↓^[^ ^266^ ^]^ IL‐1β signaling → cardiac fibrosis^[^ ^275^ ^]^	IL‐1↓→ synergize anti‐PD‐1 therapy in triple negative breast cancer^[^ ^269^ ^]^ CANTOS: canakinumab → the relative hazard for developing lung cancer↓^[^ ^270^ ^]^ CANOPY‐1: canakinumab in combination with fist‐line pembrolizumab plus chemotherapy for advanced non‐small cell lung cancer → no significant improvement^[^ ^271^ ^]^ NCT04789681 (ongoing): canakinumab for lung cancer prevention
	IL‐6	IL‐6↑→ incidence of coronary heart disease↑^[^ ^276^ ^]^ The concentration of IL‐6 is correlated with the severity of heart failure and exercise capacity^[^ ^277^ ^]^ RESCUE: ziltivekimab (IL‐6 antibody) → thrombosis↓^[^ ^280^ ^]^	IL‐6 receptor antagonist synergizes anti‐PD‐L1 antibody^[^ ^283^ ^]^ IL‐6 receptor inhibitors → immune therapy‐related adverse events↓^[^ ^284^ ^]^
CHIP	Inflammation	CHIP → risk of coronary heart disease, myocardial infarction, coronary artery calcification↑^[^ ^291^ ^]^	Cancer therapy is associated with specific accumulation of CHIP clones^[^ ^287^ ^]^ CHIP is associated with poorer prognosis among patients with solid tumor^[^ ^288^ ^]^
The mechanistic intersection of CVD and cancer in gut microbes			
Metabolites of gut microbes	TMAO	Independent predictor and promoter of atherosclerosis^[^ ^327^ ^]^ TMAO↑→ incidence of abdominal aortic aneurysm↑^[^ ^328^ ^]^ TMAO↑→ exacerbation of hypertension^[^ ^329^ ^]^	TMAO↑→ risk of colon, prostate, pancreatic, liver cancer↑^[^ ^330^, ^332^ ^]^ TMAO↑→ tumor cell proptosis and CD8+ T cells antitumor efficacy↑^[^ ^332^ ^]^ Administration of TMAO → antitumor effects in pancreatic ductal adenocarcinomas and synergizes immune checkpoints inhibitors^[^ ^333^ ^]^
	IPA	IPA↓→ risk and severity of atherosclerotic coronary artery disease↑^[^ ^334^ ^]^ Hypertension → plasma and fecal IPA↓^[^ ^335^ ^]^ IPA protects against HFpEF^[^ ^336^ ^]^	IPA↑→ responsiveness to immune checkpoint inhibitors in melanoma, breast, and colorectal cancer↑^[^ ^95^ ^]^

CVD, cardiovascular disease; LDL, low‐density lipoprotein; HDL, high‐density lipoprotein; IGF1, insulin‐like growth factor‐1; ROS, reactive oxygen species; SIRT, sirtuin; DCA, deoxycholic acid; LCA, lithocholic acid; NK, natural killer; BCAA, branched‐chain amino acid; ASCVD, atherosclerotic cardiovascular disease; CANTOS, Canakinumab Anti‐inflammatory Thrombosis Outcome Study; RESCUE, IL‐6 inhibition with ziltivekimab in patients at high atherosclerotic risk; CHIP, clonal hematopoiesis of indeterminate potential; TMAO, trimethylamine N‐oxide; IPA, indole‐3‐propionic acid; HFpEF, heart failure with preserved ejection fraction.

#### 6.1.1 Lipid Metabolism

Dyslipidemia is a significant factor that can affect both CVD and cancer. Dysregulated lipid metabolism supports cancer development at different stages. It not only promotes tumor cell proliferation but also triggers signaling and epigenetic changes that alter membrane composition, facilitating metastasis.^[^ ^176^ ^]^ An imbalance in lipid homeostasis results in lipid accumulation and cytotoxicity in cardiomyocytes. LDL transports cholesterol to peripheral tissues and accumulates within the vascular wall beneath the impaired endothelium. Oxidative stress within the subendothelial space generates oxidized LDL. Oxidized LDL gathers in macrophages, inducing proinflammatory responses and excessive macrophage apoptosis, which are pivotal for the initiation and progression of atherosclerotic lesions.^[^ ^177^ ^]^ Decreasing plasma LDL cholesterol levels lowers the incidence of cardiovascular events, with benefits remaining consistent for each unit decrease in LDL cholesterol.^[^ ^178^ ^]^ Conversely, HDL, owing to its capacity to take up free cholesterol from macrophages, facilitates reverse cholesterol transport to improve cardiovascular health.^[^ ^179^ ^]^ Although cancer risk does not show a clear, consistent relationship with either LDL or HDL cholesterol levels, clinical trials have demonstrated a positive correlation between circulating plasma cholesterol levels and mortality risk in patients with breast, ovarian, pancreatic and prostate cancer.^[^ ^180^ ^]^ Exposure to 27‐hydroxycholesterol, a circulating cholesterol metabolite that simulates conditions in hypercholesterolemic/dyslipidemic patients, enhances the capacity of cancer cells to promote tumorigenesis and metastasis.^[^ ^181^ ^]^ 27‐Hydroxycholesterol in macrophages also promotes hypercholesterolemia‐driven endothelial activation, monocyte recruitment and foam cell formation, leading to atherosclerosis.^[^ ^182^ ^]^ Inhibition of CYP27A1, an enzyme that catalyzes the conversion of cholesterol to 27‐hydroxycholesterol, has been reported to repress cancer metastasis and blunt vascular inflammation to act as a defense against atherosclerotic CVD (ASCVD), which provides a novel therapeutic target for CVD and cancer.^[^ ^182^, ^183^ ^]^

#### 6.1.2 Adipose Tissue‐Derived Adipokines

Adipose tissue, once thought to serve merely as a source of energy and insulation, is now acknowledged as a vital endocrine organ.^[^ ^184^ ^]^ It actively communicates with various organs by releasing substances such as adipokines, which supply nutrients and influence whole‐body metabolism. Many tumors (such as melanoma and pancreatic, kidney, breast and prostate cancers) are located either within or adjacent to adipose tissue, where they exert important paracrine influences and directly impact tumor proliferation and progression. Adipokines regulate atherosclerosis by modulating inflammation, as do the circulating levels of LDL and triglycerides.^[^ ^185^ ^]^ Leptin, which is secreted by adipose tissues, is a significant element in tumorigenesis and cardiovascular health. Within the physiological range of leptin levels, leptin signaling is indispensable for normal cardiovascular metabolism and contraction. However, low or high leptin levels, when they exceed physiological ranges, are correlated with short‐term detrimental cardiac events and the development of arterial stiffness in patients with CAD.^[^ ^186^ ^]^ Leptin also plays a vital role in cancer proliferation and metastasis.^[^ ^184^ ^]^ In head and neck, pancreas, cervical, and breast cancer patients, the expression level of leptin is relatively elevated compared with that in cancer‐free individuals.^[^ ^187^ ^]^ High leptin levels may predict increased hepatocellular carcinoma risk.^[^ ^188^ ^]^ Leptin stimulates several signaling pathways, including the MAPK, JAK2‐STAT3, and PI3K‐Akt pathways, to promote the proliferation, migration and invasion of cancer cells.^[^ ^189^, ^190^ ^]^

#### 6.1.3 Glucose Metabolism

Metabolic reprogramming centered on glucose metabolism is considered a characteristic of tumors.^[^ ^191^ ^]^ Evidence links a higher incidence and death rate of diabetes to various cancers, including pancreatic cancer, CRC, bladder cancer, prostate cancer and breast cancer.^[^ ^192^ ^]^ Notably, breast cancer patients tend to have elevated risks of diabetes mellitus (DM).^[^ ^193^ ^]^ Hyperglycemia is a diagnostic hallmark of DM, occurring in type 1 DM (DM1) patients due to absolute insulin deficiency and DM2 patients due to relative insulin deficiency. Warburg discovered that cancer cells preferentially obtain energy through aerobic glycolysis to turn most glucose into lactate rather than through the tricarboxylic acid cycle.^[^ ^194^ ^]^ The Warburg effect causes increased glucose uptake by cancer cells due to altered metabolism. Moreover, individuals with DM2 are at greater risk of developing CVD, as evidenced by epidemiological studies.^[^ ^195^ ^]^ DM2 is often accompanied by dyslipidemia, hypertension and obesity, all of which are established risk factors for CVD. NF‐κB, a key regulator of endothelial inflammation, plays an important role in the pathogenesis of diabetes and CVD. Hyperglycemia stimulates the upregulation of NF‐κB via advanced glycation end products and reactive oxygen species (ROS).^[^ ^196^ ^]^ NF‐κB activation induces leukocyte recruitment, adhesion molecule expression and cytokine release in diabetic cardiomyopathy.^[^ ^197^ ^]^ The NF‐κB pathway is involved in the regulation of glycolysis and respiration for energy production in both normal and cancer cells.^[^ ^198^ ^]^ NF‐κBp65, a key member of the NF‐κB family, is significantly upregulated in hepatocellular carcinoma, which promotes cancer progression by enhancing aerobic glycolysis.^[^ ^199^ ^]^ NF‐κBp65‐mediated lactate accumulation activates the PI3K/Akt signaling pathway, which further enhances cancer cell proliferation and survival. NF‐κB is also fundamental to angiogenesis, which promotes tumor growth and metastasis in cancers.^[^ ^200^ ^]^ Considering the tumor‐promoting function of NF‐κB in cancer, targeted inhibition of this pathway could be utilized in clinical treatments. Further research is needed to determine the complex role of NF‐κB, which could facilitate the development of specific inhibitors targeting NF‐κB for treating both CVD and cancer.

#### 6.1.4 Energy Metabolism

Energy metabolism plays a crucial role in both CVD and cancer, which not only controls energy production but also adjusts signaling pathways.^[^ ^201^, ^202^ ^]^ Cancer cells adapt their energy production and utilization to the dynamic alternations in the TME during cancer progression. Rapidly growing tumors have a high energy demand, resulting in the reprogramming of mitochondrial metabolism. Mitochondrial dynamics are intricately linked to cancer cell proliferation, migration and apoptosis.^[^ ^203^ ^]^ Mitochondria not only serve as the primary source of ATP during tumor progression but also play a decisive role in regulating cell death signaling and mitochondrial ROS generation.^[^ ^202^ ^]^ Owing to mitochondrial dysfunction and DNA mutations, cancer cells typically produce higher levels of ROS than normal cells do.^[^ ^204^ ^]^ ROS can drive tumorigenesis and cancer progression by causing DNA damage, inducing mutations, and activating oncogenic pathways. Indeed, the heart demands substantial energy, necessitating a constant supply of ATP to maintain its contractile function. The heart can utilize various metabolic substrates for ATP production, especially fatty acids and glucose.^[^ ^205^ ^]^ Mitochondrial metabolism provides the majority of the energy (ATP) for a healthy heart. However, in HF, increased ROS levels can damage mitochondrial DNA and function, resulting in a decrease in mitochondrial ATP production.^[^ ^206^ ^]^

Notably, NAD^+^ levels are diminished in hearts with impaired autophagy, which promotes cardiac hypertrophy and fibrosis and ultimately affects the structure and function of the heart.^[^ ^207^ ^]^ Restoring the NAD^+^/NADH ratio has emerged as a promising and clinically applicable strategy for HF treatment. Additionally, NAD^+^ is a crucial cofactor for sirtuins (SIRTs), a family of deacetylases that removes acetyl groups from histones to regulate key signaling pathways, playing crucial roles in the response to nutritional and environmental challenges such as DNA damage and oxidative stress. Emerging evidence has demonstrated the complex roles of the SIRT family in CVD and cancer. SIRT1 exerts protective effects on myocardial ischemia‒reperfusion injury by deacetylating p53 and modulating FOXO transcription factors, but the overexpression of SIRT1 may cause mitochondrial dysfunction and impair cardiac function.^[^ ^208^ ^]^ SIRT1 has been found to be overexpressed in a variety of cancers. Inactivation of the SIRT1‐FOXO3‐BNIP3 axis leads to mitochondrial dysfunction and cell death in hepatocellular carcinoma.^[^ ^209^ ^]^ SIRT3 in cardiomyocytes, an essential target for mitochondrial quality control, enhances mitochondrial function and ATP production and inhibits ROS production by deacetylating and activating various substrates (e.g., ATP5O and OPA1) to protect against cardiac hypertrophy and apoptosis.^[^ ^210^, ^211^ ^]^ SIRT3 has antitumor effects by inducing apoptosis and modulating the cell cycle to suppress cancer cell development.^[^ ^212^ ^]^ SIRT4 not only ameliorates vascular inflammation by inhibiting the NF‐κB/IκB/CXCL2/3 pathway in atherosclerosis mouse models but also suppresses tumor growth in hepatocellular carcinoma by disrupting glutamine metabolism.^[^ ^213^, ^214^ ^]^ In CVD, the induction of macrophage autophagy by SIRT6 in endothelial cells diminishes endothelial inflammation and foam cell formation, thus mitigating the progression of atherosclerosis.^[^ ^215^ ^]^ SIRT6 plays dual roles in both tumor suppression and promotion, which depend on the tumor type. SIRT6 drives prostate cancer progression through Notch signaling pathway activation, while it inhibits aerobic glycolysis to suppress the growth of several types of cancer cells.^[^ ^216^, ^217^ ^]^ These findings underscore the urgent need for systematic exploration of SIRT‐mediated molecular networks as common therapeutic targets for CVD and cancer.

Recently, mitochondrial transplantation therapy has become an intriguing area of research. Mitochondrial transfer operates as an intercellular communication mode within the vascular system. Pretransplanting exogenous mitochondria into epithelial cells to support engraftment in vascular grafts has been proposed as a treatment for ischemia and myocardial infarction.^[^ ^218^ ^]^ Exogenous mitochondria activate mitophagy through the PINK1‐Parkin signaling pathway, which enhances the engraftment ability of endothelial cells.^[^ ^218^ ^]^ In parallel, PINK1 and Parkin protein levels are dramatically increased after mitochondrial transplantation in breast cancer cells, which is associated with tumorigenesis inhibition.^[^ ^219^ ^]^ Mitophagy is hypothesized to act as an oncosuppressor that prevents malignant transformation and maintains cellular homeostasis.^[^ ^219^ ^]^ Mitochondrial transplantation can decrease cancer cell migration and increase chemotherapeutic sensitivity but does not affect the proliferation of prostate or ovarian cancer cells.^[^ ^220^ ^]^ Consequently, targeting energy metabolism, particularly with medications (metformin) or nutraceuticals (resveratrol) that increase the NAD^+^/NADH ratio by targeting the SIRT family, as well as by mitochondrial transplantation, offers an innovative therapeutic strategy for both CVD and cancer, aiming to disrupt cancer cell growth and improve energy utilization in CVD.

#### 6.1.5 Bile Acid Metabolism

Bile acids (BAs), which are synthesized from cholesterol in the liver, initially emerge as amphipathic surfactants^[^ ^221^ ^]^. In recent years, BAs have been regarded mainly as systemic endocrine hormones, and their crucial roles in regulating macronutrient (lipid, carbohydrate, protein) metabolism and proinflammatory/anti‐inflammatory balance have attracted extensive attention.^[^ ^222^ ^]^ BAs depend on enterohepatic circulation to maintain BA pool stability and regulate energy metabolism. The gut microbiota along the gastrointestinal tract metabolizes BAs and shapes the BA pool.^[^ ^223^ ^]^ Microbiota‐dependent BAs have long been recognized to contribute to carcinogenesis,^[^ ^224^ ^]^ particularly CRC^[^ ^225^ ^]^ and hepatocellular carcinoma.^[^ ^226^, ^227^ ^]^ Collins et al.^[^ ^228^ ^]^ reviewed and summarized the main carcinogenic mechanisms of primary and secondary BAs, the latter of which are more carcinogenic because of their hydrophobicity. Similarly, the gut microbiota functions as a link between BAs and cardiovascular health. Hydrophobic BAs also play damaging roles in CVD, manifesting as cardiotoxicity. Intestinal dysbiosis can increase cardiotoxic BA levels, and increased BA levels significantly affect the metabolism and function of cardiomyocytes, vascular endothelial cells and vascular smooth muscle cells, leading to hypertension, atherosclerosis, unstable angina, and HF.^[^ ^229^ ^]^ Notably, hydrophilic BAs, such as ursodeoxycholic acid (UDCA), are usually cardioprotective, preventing reentrant arrhythmias and protecting against reperfusion injury.^[^ ^230^, ^231^ ^]^ The dual role of BAs in CVD reflects the complexity of BA metabolism. Interestingly, BAs also coordinate with the gut microbiome to modulate immunological homeostasis, thereby influencing CVD and cancer.^[^ ^232^ ^]^ For example, the gut microbiome uses BAs as messengers to control CXCL16‐dependent accumulation of hepatic natural killer T cells and anticancer immunity in the liver, where primary BAs increase CXCL16, whereas secondary BAs reduce it.^[^ ^233^ ^]^ A recent study by Varanasi et al. highlighted BA‐mediated regulation of immunosurveillance and cancer progression in the liver.^[^ ^234^ ^]^ The accumulation of primary conjugated and secondary BAs is a metabolic feature of HCC, in which immunotoxic primary BAs induce oxidative stress and T‐cell death, whereas immunosuppressive secondary BAs activate endoplasmic reticulum stress to promote T‐cell dysfunction.^[^ ^234^ ^]^ For CVD, particularly cardiometabolic disease, BAs can either increase or inhibit inflammatory responses to shape systemic immunometabolism, which has been systematically reviewed by Guan et al.^[^ ^235^ ^]^ Undoubtedly, BA homeostasis is crucial for both CVD and cancer and is a potential therapeutic target. However, much work remains to be done to identify complex and dynamic BAs, including identifying more BA species and elucidating the diverse functions of BAs.

#### 6.1.6 Ceramide Metabolism

Alterations in the BA pool composition directly influence the balance of ceramide synthesis and catabolism, the metabolism of which also contributes to the progression of both CVD and cancer.^[^ ^236^ ^]^ Ceramide is the structural building block of sphingolipids and consists of a sphingosine backbone and fatty acid chain of varying length. It has become a focus of research in the fields of cardiology and oncology, as it functions not only as a structural component of the plasma membrane but also as a crucial signaling molecule that regulates cell proliferation, differentiation, and apoptosis. Increased plasma ceramide levels are associated with atherosclerosis and HF.^[^ ^237^ ^]^ Clinical scores, including the coronary event risk test 1 (CERT1) and CERT2 scores, which are based on ceramides, have been developed to stratify the risk of ASCVD. CERT1 is based on four specific ceramides and their ratios, whereas CERT2 incorporates additional phosphatidylcholines to increase its predictive accuracy.^[^ ^238^ ^]^ Recently, it was demonstrated that the CYSLTR2 and P2RY6 receptors are directly bound and activated by ceramide, facilitating atherosclerotic plaque formation.^[^ ^239^ ^]^ These findings provide novel therapeutic targets for ameliorating the detrimental effects of ceramide. In patients with lipotoxic cardiomyopathy and ischemic cardiomyopathy, myocardial ceramide levels are elevated.^[^ ^240^ ^]^ The absence of myocardial Nogo‐A, which is a negative regulator of serine palmitoyltransferase (SPT), a rate‐limiting enzyme for ceramide *de novo* synthesis, contributes to ceramide accumulation in the heart, leading to more severe cardiac hypertrophy and dysfunction.^[^ ^240^ ^]^ In the field of oncology, ceramide has been intensively investigated for its role in regulating apoptosis, as it is induced by chemotherapy and radiotherapy to mediate cancer cell death.^[^ ^241^ ^]^ However, ceramide can also promote cancer progression and contribute to remodeling of the TME. C_24_‐Ceramide serves as a potential biomarker for gallbladder cancer, facilitating its early diagnosis.^[^ ^242^ ^]^ Additionally, C_24_‐ceramide promotes the formation and activation of the mTOR complex, thereby enhancing the proliferation and migration of gallbladder cancer cells.^[^ ^242^ ^]^ In the TME, myeloid‐specific deficiency of neutral ceramidase, which is responsible for hydrolyzing ceramide into sphingosine, can polarize macrophages into immunosuppressive TREM2^+^ tumor‐associated macrophages (TAMs) and then promote the exhaustion phenotype of CD8^+^ T cells to drive breast cancer progression.^[^ ^243^ ^]^ Ceramide‐related translational studies have been conducted, but their therapeutic efficacy remains uncertain. Fenretinide, a retinoid with established anticancer activity, functions as an inhibitor of dihydroceramide desaturase 1 (DES1), an enzyme responsible for catalyzing the final step in *de novo* ceramide synthesis. Inhibiting this pathway is expected to impede the progression of atherosclerosis by reducing the production of deleterious ceramide. However, in preclinical studies, fenretinide administration unexpectedly accelerated atherosclerosis development.^[^ ^244^ ^]^ These unsatisfactory results may stem from its retinoid activity rather than its DES1 inhibition, underscoring the need for novel therapeutic targets and the development of more selective medications. Since ceramide plays a significant modulatory role in the pathogenesis and progression of both CVD and cancer, regulating ceramide homeostasis represents a promising therapeutic direction, with the discovery of more precise targets and the development of novel therapeutics facilitating the translation of ceramide‐related basic research.

#### 6.1.7 Branched‐Chain Amino Acid Metabolism

Recent studies in oncology and cardiology have increasingly focused on branched‐chain amino acid (BCAA) metabolism because of its critical roles in energy production, signaling pathways (such as mTOR), and its association with disease progression and therapeutic potential. BCAAs, including leucine, isoleucine, and valine, are essential amino acids that cannot be synthesized by the human body. BCAA metabolism is initiated by BCAA transaminases (BCAT), which convert BCAA into branched‐chain keto acid (BCKA). This is followed by a rate‐limiting dehydrogenation step catalyzed by BCKA dehydrogenases.^[^ ^245^ ^]^ Human‐based data suggest that increased plasma levels of BCAAs are correlated with an increased risk of pancreatic cancer,^[^ ^246^ ^]^ lung cancer,^[^ ^247^ ^]^ atherosclerosis,^[^ ^248^ ^]^ thrombosis,^[^ ^249^ ^]^ and HF.^[^ ^250^ ^]^

Increased plasma BCAA levels mediated by high dietary intake or the inhibition of BCAA metabolic enzymes promote the progression of atherosclerosis.^[^ ^248^ ^]^ Mechanistically, BCAA‐mediated mitochondrial elevation of H_2_O_2_ promotes the translocation of high mobility group box 1 (HMGB1) to the extracellular space, where it binds to Toll‐like receptor 4 (TLR4) to further drive the inflammatory transition of macrophages.^[^ ^248^ ^]^ In addition, BCAAs increase the propionylation of tropomodulin‐3 in platelets, thereby increasing platelet activity and promoting thrombosis formation.^[^ ^249^ ^]^ These findings indicate that elevated BCAA levels play a significant pathogenic role in CHD by amplifying inflammation and thrombosis‐related processes. BCAAs are intimately involved in the development of cardiomyopathy and HF. Low‐BCAA diets lead to reduced plasma BCAA levels, improve post‐MI cardiac systolic function, enhance early fibrotic repair, and attenuate the inflammatory response.^[^ ^251^ ^]^ Impaired catabolic capacity of the gut microbiota in type 1 diabetic mice leads to elevated plasma BCAA levels, which subsequently suppress hepatic fibroblast growth factor 12 expression, thereby increasing cardiac BCAA accumulation via the upregulation of cardiac BCAA receptors.^[^ ^252^ ^]^ Accumulation of cardiac BCAAs induces mitochondrial damage and myocardial apoptosis, leading to diabetic cardiomyopathy.^[^ ^252^ ^]^ BCAAs exacerbate HF by impairing mitochondrial respiration and ATP synthesis and by increasing peripheral blood pressure.^[^ ^253^, ^254^ ^]^ Furthermore, in patients with HFpEF, myocardial BCAA levels are elevated compared with those in nonfailing controls due to impaired BCAA catabolism.^[^ ^255^ ^]^ Increased cardiac BCAA levels contribute to myocardial lymphatic defects in mice with HFpEF. These lymphatic defects are significant because they impair the drainage of interstitial fluid and inflammatory agents, which is essential for maintaining cardiac homeostasis.^[^ ^250^ ^]^ Increased BCAA catabolism helps preserve lymphatic integrity and offers protection against HFpEF.^[^ ^250^ ^]^

Similarly, dysregulated BCAA metabolism has also been implicated in cancer development and progression. For example, BCAT2, which is expressed in mitochondria, is elevated in mouse and human pancreatic ductal carcinoma cells.^[^ ^256^ ^]^ Pancreatic‐specific knockout of *Bcat2* or administration of a BCAT2 inhibitor precludes the progression of pancreatic intraepithelial neoplasia.^[^ ^256^ ^]^ A gain‐of‐function genetic mutation in cytoplasmic BCAT1 enhances metabolic ability and promotes the proliferation and motility of lung and gastric cancer cells in a RhoC‐dependent manner.^[^ ^257^ ^]^ Collectively, restricting daily BCAA intake may represent a cost‐effective and promising strategy for the treatment of cancer and CVD. BCAAs and their metabolic byproducts influence the development of CVD and cancer through multiple mechanisms, including dysregulation of signaling pathways and promotion of inflammation. Targeting BCAA metabolism may represent a promising therapeutic strategy for the treatment of both diseases.

By delving into metabolic regulation, it has been elucidated that multiple metabolic abnormalities can concurrently affect both CVD and cancer, revealing putative shared therapeutic mechanisms. This finding also suggests that clinicians should be vigilant with respect to the occurrence of both CVD and cancer when encountering abnormal metabolic indicators in clinical trials.

### 6.2 Immunoinflammation Justifies Common Immunotherapy for CVD and Cancer

#### 6.2.1 Macrophages

**Figure 4:**
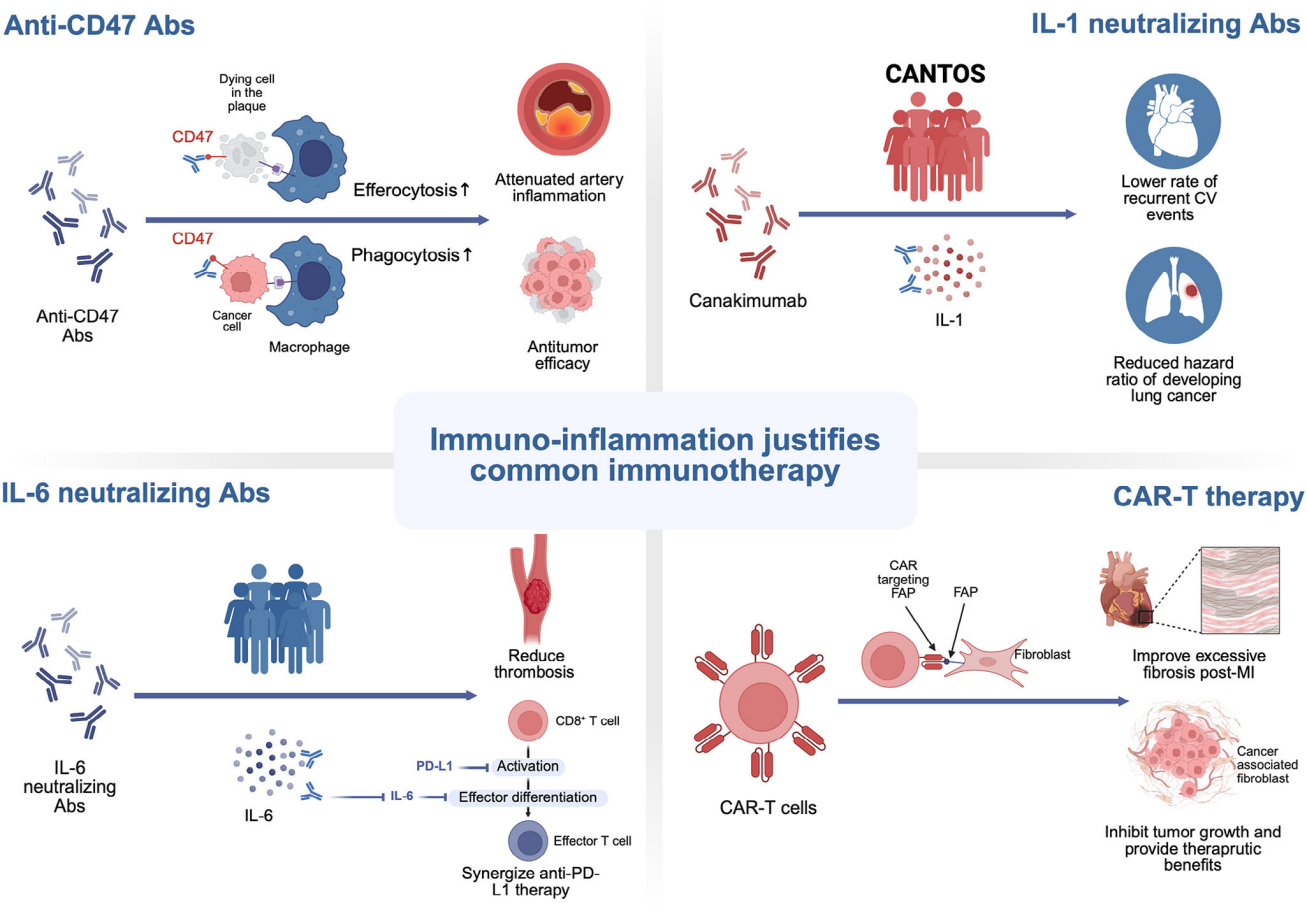
Immunoinflammation is a common immunotherapy for CVD and cancer. Intersectional immune‐inflammation mechanisms, such as the involvement of macrophages and interleukins in the pathogenesis of CVD and cancer, suggest that immunotherapy could serve as a prospective homotherapy for both diseases. CANTOS, Canakinumab Anti‐inflammatory Thrombosis Outcome Study; FAP, fibroblast activation protein; Abs, antibodies; CV, cardiovascular; MI, myocardial infarction; CAR, chimeric antigen receptor. Created with BioRender.com.

Macrophages are heavily involved in cancer progression and CVD, particularly atherosclerosis. In atherosclerosis, macrophages are recruited, adhere, and transmigrate into the subintimal space, where they ingest oxidized lipoproteins to form foam cells, the hallmark pathological feature of atherosclerosis. In the early stage of atherosclerosis, macrophages help curb the expansion of the necrotic core and the progression of plaques through efferocytosis.^[^ ^258^ ^]^ However, in advanced atherosclerosis, apoptotic cell clearance is impaired not only by the diminished phagocytotic ability of macrophages but also by the upregulation of “don't eat me” molecules (e.g., CD47, a myeloid checkpoint) on dying cells.^[^ ^259^ ^]^ In a retrospective analysis, it was unexpectedly found that patients treated with magrolimab, a CD47‐targeting macrophage checkpoint inhibitor that is administered in combination with rituximab to treat lymphoma, presented attenuated carotid artery inflammation, suggesting potential enhancement of efferocytosis‐mediated inflammation resolution.^[^ ^260^ ^]^ In parallel, various cancer cells express CD47 to protect them from phagocytosis by macrophages.^[^ ^261^ ^]^ CD47 is highly expressed in the peritoneal carcinomatosis of gastric adenocarcinoma and is inversely associated with prognosis.^[^ ^262^ ^]^ In preclinical models of small cell lung cancer, a highly metastatic cancer, the combination of radiotherapy and CD47 blockade not only enhances local antitumor efficacy but also exerts remote antitumor effects dependent on activated M1‐like macrophages (**Figure** **4**).^[^ ^263^ ^]^ Notably, statins, the first‐line medication for ASCVD, possess a novel function in enhancing macrophage‐mediated efferocytosis.^[^ ^264^ ^]^ Statins can inhibit the prenylation and activation of RHOA, an effective inhibitory protein, by diminishing cholesterol synthesis and constraining the translocation of NF‐κB to the nucleus, thereby restraining CD47 upregulation and exerting an additive effect when combined with anti‐CD47 antibodies.^[^ ^259^ ^]^ However, it is not yet fully understood whether statins enhance the efficacy of anti‐CD47 antibodies in cancer treatment through increased efferocytosis. Although the effects of anti‐CD47 therapy in atherosclerosis require further validation in prospective studies, macrophages and myeloid checkpoint inhibitors are prospective homotherapeutic candidates for treating cancer and CVD.

#### 6.2.2 Interleukins

Interleukins function as key orchestrators of intracellular communication and precise regulators of inflammation and have long been hypothesized to play a critical role in the pathogenesis of atherosclerosis. Interleukin‐1 (IL‐1) has great potential as a target for intervention in the pathogenesis of atherosclerosis.^[^ ^265^ ^]^ The Canakinumab Anti‐inflammatory Thrombosis Outcome Study (CANTOS), a randomized, double‐blinded, placebo‐controlled trial, demonstrated that the administration of a monoclonal antibody targeting IL‐1β—canakinumab—significantly lowered the rate of recurrent cardiovascular events.^[^ ^266^ ^]^ In oncology, IL‐1 facilitates both epithelial and mesenchymal primary oncogenesis, drives the development of an immunosuppressive TME, and accelerates tumor progression.^[^ ^267^, ^268^ ^]^ From a therapeutic perspective, neutralizing IL‐1β during the early stages of tumor development alleviates immunosuppression in the TME and further synergizes with anti‐PD‐1 therapy to inhibit tumor growth in mouse models of triple‐negative breast cancer.^[^ ^269^ ^]^ In humans, patients in the treatment arm of the CANTOS trial experienced a remarkable 67% reduction in the relative hazard of developing lung cancer.^[^ ^270^ ^]^ Nevertheless, in a trial comparing canakinumab with a placebo, both combined with first‐line pembrolizumab plus chemotherapy for advanced non–small cell lung cancer, no significant improvement in progression‐free or overall survival was observed.^[^ ^271^ ^]^ Subgroup analyses of PD‐L1, tumor mutation burden, CRP, and IL‐6 have already been conducted; however, further analyses, such as those incorporating immune cell infiltration, blood profiling of IL‐1β, the neutrophil‐to‐lymphocyte ratio, and other factors, are still needed to identify patients who could benefit from canakinumab.^[^ ^271^ ^]^ A clinical trial evaluating the efficacy of canakinumab in inducing the regression of pulmonary nodules in patients at high risk of non‐small cell lung cancer (ClinicalTrials.gov identifier: NCT04789681) is still ongoing. Considering the critical role of IL‐1 in the initiation and progression of CVD and cancer, as well as findings from the aforementioned randomized controlled trials, it is rational to anticipate the homotherapeutic efficacy of targeting IL‐1 for CVD and cancer.^[^ ^272^, ^273^, ^274^, ^275^ ^]^

In addition to IL‐1, IL‐6 plays a pivotal role in the pathogenesis of both CVD and cancer, acting as a key mediator of innate and adaptive immune responses by facilitating the expansion and activation of T cells, promoting the differentiation of B cells, and regulating acute‐phase responses.^[^ ^276^ ^]^ A single‐nucleotide polymorphism that results in increased expression of IL‐6 is associated with an increased incidence of CHD.^[^ ^276^ ^]^ The concentration of IL‐6 is elevated in patients with heart HF regardless of whether they have a reduced or preserved ejection fraction, and it is correlated with the severity of HF and exercise capacity.^[^ ^277^ ^]^ In a CANTOS trial substudy, IL‐6 levels were reported to be downregulated alongside IL‐1 levels following the administration of canakinumab and were causally associated with a lower incidence of cardiovascular events.^[^ ^278^ ^]^ In contrast, anti‐inflammatory treatment without reducing IL‐6 through low‐dose methotrexate failed to reduce cardiovascular events, which emphasizes the importance of IL‐6 in anti‐inflammatory therapies.^[^ ^279^ ^]^ The administration of ziltivekimab, a monoclonal antibody targeting IL‐6, significantly reduced the levels of inflammation biomarkers, such as hypertensive C‐reactive protein (CRP), and thrombosis in a randomized, double‐blinded trial involving high cardiovascular risk patients.^[^ ^280^ ^]^ To identify a more specific pharmacological target to mitigate the side effects caused by IL‐6 inhibition, Prapiadou et al. determined that CXCL10 mediates most of the atheroprotective effects resulting from the downregulation of IL‐6 signaling.^[^ ^281^ ^]^ The role of IL‐6 in tumorigenesis is complex and paradoxical. While exercise‐stimulated transient release of IL‐6 from skeletal muscle has multiple benefits, including increased production of anti‐inflammatory cytokines, reduced proliferation and DNA damage in cancer cells, and enhanced antitumor immune responses, constant elevation of IL‐6 shifts inflammation from an acute phase to a chronic phase, thus promoting the malignant transformation of normal cells and creating a favorable TME for tumor progression and metastasis.^[^ ^282^ ^]^ The duration of IL‐6 exposure, the signaling mode, the upstream modulator, and the cellular source of IL‐6 may all contribute to its dual effects on cancer.^[^ ^282^ ^]^ Additionally, atezolizumab (an anti‐PD‐L1 antibody) has shown relatively poor clinical outcomes in large clinical trials for advanced kidney, breast, and bladder cancers. Combining PD‐L1 therapy with IL‐6 antagonists has potential for improved efficacy.^[^ ^283^ ^]^ In a multicenter retrospective analysis, improvements in immune‐related adverse events (irAEs), key contributors to the interruption or discontinuation of cancer treatment, were evaluated following the administration of immune checkpoint therapy and IL‐6 receptor antagonists (tocilizumab or sarilumab).^[^ ^284^ ^]^ Targeting the IL‐6 receptor has been reported as a promising strategy to combat irAEs while preserving antitumor immunity. In brief, targeting IL‐6 may serve as another promising homotherapeutic approach for both CVD and cancer by modulating immune responses and inflammation (Figure 4).

#### 6.2.3 Clonal Hematopoiesis of Indeterminate Potential

Clonal hematopoiesis of indeterminate potential (CHIP), an age‐associated, premalignant condition, is characterized by the accumulation of mutated hematopoiesis clones (e.g., *DNMT3A‐, TET2‐*, and *JAK‐*mutated clones) that retain the ability to differentiate into leukocytes.^[^ ^285^, ^286^ ^]^ Cancer therapies for patients with solid tumors, such as chemotherapy and radiotherapy, are associated with the accumulation of specific CHIP clones, including *TP53, PPM1D*, and *CHEK2*.^[^ ^287^ ^]^ Despite the higher risk of hematopoietic malignancy, CHIP is correlated with poorer overall survival among patients with solid tumors, especially those with higher variant allele fractions, even after adjusting for age, smoking status, and sex.^[^ ^288^ ^]^ Shorter overall survival is not due to hematopoietic malignancy; instead, it is caused primarily by the progression of primary nonhematologic tumors. CHIP is linked to an elevated inflammatory state, as indicated by increased serum levels of IL‐1β, IL‐6, and IFN‐γ in individuals with *TET2, JAK, and DNMT3A* mutations, respectively.^[^ ^289^ ^]^ In parallel, patients with *TET2* mutations, characterized by enhanced IL‐1β signaling, demonstrated a more pronounced response to canakinumab in preventing major cardiovascular adverse events during the CANTOS trial.^[^ ^290^ ^]^ Additionally, CHIP carriers have a 1.9‐fold increased risk of CHD, a 4.0‐fold greater risk of myocardial infarction, and a greater coronary artery calcification burden than noncarriers do.^[^ ^291^ ^]^ In a mouse model lacking *TET2*—the second most common CHIP mutation—in the myeloid lineage, *Il1b* and *Il6* gene expression was significantly upregulated in macrophages.^[^ ^291^ ^]^ Therefore, it is plausible that an anti‐inflammatory regimen targeting interleukin modulation may provide dual benefits in cardiology and oncology because of the close associations among CHIP, cancer, and CVD.

#### 6.2.4 Chimeric Antigen Receptor T‐Cell Therapy

Notably, despite some homotherapeutic candidates based on intersectional immune mechanisms, treatments utilizing common immune technologies continue to be developed (Figure 4). Chimeric antigen receptor (CAR)‐T‐cell therapy, a form of adoptive cell therapy, has achieved remarkable success in treating hematological malignancies and is currently undergoing clinical trials to assess its efficacy against solid tumors.^[^ ^292^ ^]^ CAR‐T‐cell therapy also shows potential as a strategy to combat heart disease. Fibrosis is an integral component of tissue repair, but hyperactivated fibroblasts and excessive fibrosis reduce cardiac compliance and increase heart stiffness. However, effective interventions to address excessive fibrosis and enhance cardiac function remain scarce for patients with impaired cardiac compliance and relaxation, which are key contributors to HF.^[^ ^293^, ^294^ ^]^ Fibroblast activation protein (FAP) expression is significantly upregulated in the cardiac fibroblasts of patients with hypertrophic or dilated cardiomyopathy compared with healthy controls, making FAP a promising candidate for targeting activated fibroblasts to modulate pathological fibrosis.^[^ ^294^ ^]^ Rurik et al. developed modified mRNA encoding a CAR targeting FAP, encapsulated it in T‐cell‐targeted lipid nanoparticles (LPNs), and successfully generated transient CAR‐T cells in vivo by delivering mRNA‐loaded LPNs, which led to notable functional improvement in mice with injured and fibrotic hearts.^[^ ^295^ ^]^ Notably, FAP expression is elevated in various cancers and is frequently used as a marker of protumorigenic stroma.^[^ ^296^ ^]^ Targeting FAP in the tumor stroma with CAR‐T cells inhibits tumor growth and provides therapeutic benefits.^[^ ^297^ ^]^ Additionally, the contrasting expression pattern of FAP—low in healthy tissues but elevated in most malignant epithelial tumors, inflammatory lesions, and atherosclerosis—enables the use of FAP‐targeted molecular imaging to simultaneously visualize tumors and assess atherosclerosis progression.^[^ ^298^ ^]^

### 6.3 Gut Microbes Rationalize Microbe/Metabolite‐Targeted Homotherapy for CVD and Cancer

#### 6.3.1 Gut Microbiota

**Figure 5:**
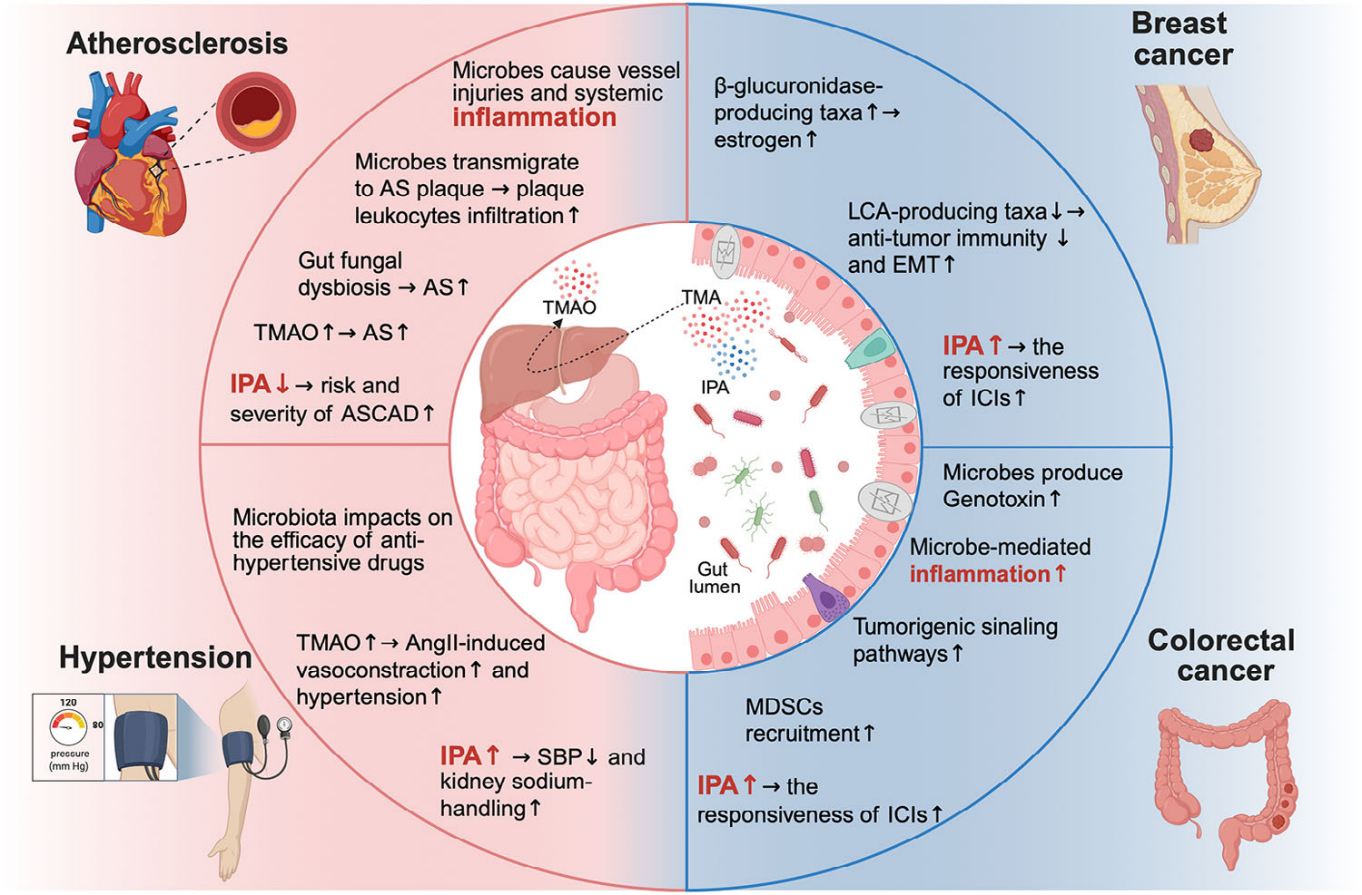
Gut microbes rationalize microbe/metabolite‐targeted homotherapy for CVD and cancer. Dynamic alterations in the gut microbiome are associated with the initiation and progression of CVD and cancer. The beneficial or harmful influences exerted by the gut microbiota and their metabolites converge, providing a rationale for microbe‐ or metabolite‐targeted homotherapy for CVD and cancer. Potential homotherapeutic targets are highlighted in bold, magnified, and presented in red. AS, atherosclerosis; ASCVD, atherosclerotic cardiovascular disease; TMA, trimethylamine; TMAO, trimethylamine N‐oxide; IPA, indole propionate acid; SBP, systolic blood pressure; LCA, lipopolysaccharide; EMT, endothelial‒mesenchymal transition; ICIs, immune checkpoint inhibitors; MDSCs, myeloid‐derived suppressor cells. Created with BioRender.com.

Gut microbes are microorganisms that reside in the gastrointestinal tract, including bacteria, fungi, viruses, etc. Gut microbes have evolved into symbiotic relationships where humans provide them with nutrients, and in return, humans can benefit from their presence, such as through the production of vitamins B and K by microbes. Dynamic alterations in the gut microbiome are associated with organ homeostasis and the initiation and progression of diseases, including CVD and cancer (**Figure** **5**).^[^ ^299^, ^300^, ^301^ ^]^

Alterations in the gut microbiota have been linked to atherosclerosis from several perspectives. First, studies have shown that patients with atherosclerosis have a greater abundance of *Enterobacteriaceae* and *Streptococcus spp*. in their gut microbiome compared to healthy controls.^[^ ^302^ ^]^ *Streptococcus* spp. are consistently overrepresented in individuals with atherosclerosis, regardless of whether the condition is clinical or subclinical. These findings have been further validated by studies focusing on populations with subclinical atherosclerosis, which minimize the potential biases introduced by anti‐atherogenic medications.^[^ ^303^ ^]^ Mechanistically, the gut microbiome facilitates the initiation or progression of atherosclerosis by directly sparking vessel injuries or indirectly inducing systemic inflammation through local or distant infections, thus mediating lipid metabolism and dietary metabolites.^[^ ^304^ ^]^ The gastrointestinal lumen, serving as a reservoir for microbes, may be one of the origins of plaque‐residing microbes. Potentially pathogenic microbial taxa are more abundant in the atherosclerotic plaques of symptomatic patients than in those of asymptomatic patients.^[^ ^304^ ^]^ Additionally, plaque microbiota are positively associated with the number of plaque‐infiltrating leukocytes, suggesting that plaque‐residing microbiota may exacerbate the in situ inflammatory response.^[^ ^305^ ^]^ In addition, although their abundance is lower than that of bacteria, intestinal fungi have been reported to be involved in atherosclerosis. Gut fungal dysbiosis emerges in the early stages of atherosclerosis and is characterized by an increased abundance of *Candida albicans* in patients with dyslipidemia compared with healthy individuals.^[^ ^306^ ^]^ Elevated levels of *Candida albicans* upregulate intestinal hypoxia‐inducible factor 2α (HIF‐2α), which in turn enhances ceramide synthesis, thereby promoting the progression of atherosclerosis.^[^ ^306^ ^]^ The mechanisms underlying hypertension are explained by the mosaic theory of hypertension, which, in its newest version from 2021, introduced the microbiome as one of the key contributors.^[^ ^307^ ^]^ Microbiome dysbiosis has been confirmed in patients with hypertension and in rat models of spontaneous hypertension.^[^ ^308^ ^]^ Fecal microbiota transplantation from hypertensive and normotensive patients to germ‐free mice was conducted to investigate the role of gut microbes in hypertension. The results revealed that recipient mice harboring microbiota from hypertensive patients presented greater blood pressure than controls harboring microbiota from normotensive subjects did, suggesting that the gut microbiota modulates blood pressure directly or indirectly via metabolites.^[^ ^309^ ^]^ In addition, the gut microbiota influences the response to antihypertensive medications. For example, losartan, an Ang II receptor blocker, reduces dysbiosis and restores gut integrity in spontaneously hypertensive rats, which contributes, at least in part, to the efficacy of losartan.^[^ ^310^ ^]^ *Coprococcus* reduces the blood pressure‐lowering effects of quinapril, an ACEI, as *C*. can catabolize esters.^[^ ^311^ ^]^

Accumulating evidence has shown that the gut microbiome can fuel oncogenesis and cancer progression.^[^ ^312^, ^313^ ^]^ CRC is among the most prevalent cancers of the digestive tract. The gastrointestinal tract is the direct habitat of gut microbes, and the oncogenesis and progression of CRC are closely related to gut microbes.^[^ ^314^ ^]^ Mechanistically, microbes promote the pathogenesis of CRC through various pathways. Genotoxins produced by the gut microbiota lead to oncogenic mutagenesis. Cytolethal distending toxin (CDT), produced by *Campylobacter jejuni*, which abounds in patients with CRC, mediates DNA double‐strand breaks through DNase bioactivity. The introduction of *C. jenuni*, which prevents the production of functional CDT in mice, abolishes its tumorigenic effects.^[^ ^315^ ^]^ Similarly, the DNA‐damaging and tumorigenic effects of colibactin and indolimine produced by *E. coli* and *M. morganii*, respectively, are corroborated by loss‐of‐function tests, as demonstrated by the attenuation of CRC following the blockade of colibactin synthesis or the introduction of nonindolimine‐producing *M. morganii*.^[^ ^314^, ^316^ ^]^ Inflammation is a critical factor in tumorigenesis, in which gut microbes also play a key role in triggering the inflammatory response. For example, enterotoxigenic *B. fragilis* triggers inflammation by disrupting gut barrier function and activating the Th17 cell‐dependent inflammatory cascade, whereas blockade of IL‐17 signaling effectively reduces inflammation and CRC development in mouse models.^[^ ^314^ ^]^ Additionally, colonization by certain microbes (*F. nucleatum, P. anaerobius*, etc.) results in the recruitment of myeloid‐derived suppressor cells to inhibit the antitumor activities of T cells.^[^ ^314^ ^]^ Interestingly, remote organs that are not physically connected to the gastrointestinal tract are also influenced by the gut microbiota. For example, in the gut of breast cancer patients, the abundances of *Porphyromonas* and *Peptoniphilus* increased, whereas the abundances of *Odoribacter sp*., *Butyricimonas sp*., and *Coprococcus sp*. decreased, and the gut microbiota diversity decreased.^[^ ^317^ ^]^ Increased estrogen levels, a high‐risk factor for breast cancer, may be a consequence of reduced microbiota diversity, resulting in an increase in β‐glucuronidase‐producing taxa, which release estrogen from glucuronide‒estrogen conjugates.^[^ ^318^ ^]^ Lithocholic acid (LCA), a secondary BA produced by gut microbes, is downregulated in patients with breast cancer. A lower concentration of LCA is associated with a poorer prognosis in patients with breast cancer, as LCA induces oxidative phosphorylation, which inhibits epithelial‒mesenchymal transition (EMT) and stimulates antitumor immunity.^[^ ^319^ ^]^ In parallel with the intensive investigations of fungi in the pathogenesis of CVD, fungi have also been increasingly studied in cancer. Ablation of intestinal fungi with antifungal agents enhances the efficacy of radiation therapy.^[^ ^320^ ^]^ Intestinal fungi influence the response of breast cancer to radiation therapy through intratumoral Dectin‐1, an innate immune receptor that senses fungi, and deficiency of this receptor mimics the effects of intestinal fungal ablation.^[^ ^320^ ^]^

Given the critical role of the gut microbiome in CVD and cancer, modulating the microbial composition through the administration of antibiotics, probiotics, prebiotics, and dietary interventions may emerge as a prospective approach for homotherapy in patients with both CVD and cancer. The use of minocycline has successfully restored the *Firmicutes/Bacteroidetes* (F/B) ratio, which is a biomarker for dysbiosis, resulting in increased diversity of the gut microbiome and shifting microbial metabolite production in a beneficial direction, thereby illuminating, at least in part, the antihypertensive effects of minocycline.^[^ ^308^ ^]^ Notably, minocycline has tumor‐suppressive effects on CRC by inhibiting EMT and cancer cell metastasis, although these effects are mediated by the LYN‐STAT3 axis in cancer cells but not by modifications to gut microbes.^[^ ^321^ ^]^ To some extent, compared with antibiotics, probiotics target the abundance of certain microbes more precisely. Probiotics are live microorganisms that, when administered in adequate amounts, confer health benefits to the host.^[^ ^322^ ^]^ *R. intestinalis*, a probiotic, and its metabolite butyrate can antagonize the pathogenic role of neutrophils, inflammation, and phenotype switching of vascular smooth muscle cells in abdominal aortic aneurysms (AAAs), thereby ameliorating AAA.^[^ ^323^ ^]^ Administration of *Lactobacillus murinus* can not only improve both the systolic and diastolic blood pressure in salt‐sensitive mice but also hinder tumor progression by stimulating the innate immune response, indicating that Lactobacillus murinus has therapeutic potential for patients suffering from CVD and cancer simultaneously.^[^ ^324^, ^325^ ^]^ Despite the pathogenic effects of some gut microbes, they are anticipated to act as noninvasive tools to facilitate the early diagnosis of diseases.^[^ ^314^ ^]^ For example, specific alterations in the gut microbiota of patients with CRC have facilitated the development of microbial biomarkers for screening and early detection of CRC.^[^ ^326^ ^]^ Furthermore, targeted antibiotics, phage‐based therapies, and small‐molecule strategies are expected to optimize therapeutic interventions for the gut microbiome.^[^ ^312^ ^]^

#### 6.3.2 Gut Microbiota‐Derived Metabolites

Additionally, metabolites of gut microbes have varying impacts on the pathogenesis of CVD and cancer. Trimethylamine (TMA) is a degradation product of phosphatidylcholine produced by gut microbes, and it is further oxidized by liver flavin monooxygenase to trimethylamine N‐oxide (TMAO), which is an independent predictor and promoter of atherosclerosis.^[^ ^327^ ^]^ Plasma concentrations of TMAO are elevated in patients with AAA and are positively correlated with larger infrarenal aortic diameters.^[^ ^328^ ^]^ The activation of the protein kinase RNA‐like endoplasmic reticulum kinase (PERK)‐mediated unfolded protein response (UPR) is suggested to mediate the deterioration of AAA.^[^ ^328^ ^]^ TMAO also has synergistic effects with Ang II‐induced vasoconstriction and further exacerbates hypertension.^[^ ^329^ ^]^ In oncology, TMAO is associated with increased risks of various cancers, including colon, prostate, pancreatic, and liver cancer, which may be attributed to increased expression of inflammatory genes and increased production of ROS.^[^ ^330^, ^331^ ^]^ However, TMAO‐activated PERK induces gasdermin E‐mediated pyroptosis in tumor cells and enhances the CD8^+^ T‐cell‐mediated antitumor effects on triple‐negative breast cancer (TNBC) in vivo.^[^ ^332^ ^]^ In parallel, the administration of TMAO to orthotopic pancreatic ductal adenocarcinoma (PDAC) models confers antitumor effects in a type I interferon‐dependent manner and enhances the efficacy of ICIs when used in combination.^[^ ^333^ ^]^ Taken together, the role of TMAO in the initiation and progression of cancer requires further investigation because of its complicated effects, with the aim of determining whether TMAO antagonists can be used as prophylactic agents for preventing both CVD and cancer.

Another gut microbe metabolite, indole‐3‐propionic acid (IPA), which is a tryptophan metabolite exclusively produced by gut microbes, plays an important role in the pathogenesis of CVD and cancer. A decreased abundance of IPA‐producing *Clostridium* and *Peptostreptococcus*, along with reduced plasma levels of IPA, has been reported in patients with CAD.^[^ ^334^ ^]^ Reduced plasma levels of IPA are associated with the risk and severity of atherosclerotic CAD.^[^ ^334^ ^]^ Mechanistically, IPA downregulates the expression of miR‐142‐5p, which in turn elevates ATP‐binding cassette transporter A1 (ABCA1) expression, thereby increasing cholesterol efflux from macrophages to apolipoprotein A‐I. In mice with L‐arginine methyl ester hydrochloride and high‐salt diet‐induced hypertension, decreased levels of IPA are detected in the plasma and fecal samples compared with normotensive mice, along with reduced levels of Tregs and increased Th17 cells in the kidney. Supplementation with IPA shifted the levels of Tregs and Th17 cells toward those observed in normotensive mice, leading to improved systolic blood pressure and increased sodium‐handling capacity.^[^ ^335^ ^]^ HFpEF is a highly heterogeneous disease for which effective therapy is lacking. Supplementation with IPA attenuates diastolic dysfunction and improves metabolic homeostasis of the heart by suppressing nicotinamide N‐methyl transferase (NNMT) and restoring NAD^+^/NADH levels, as well as SIRT3 levels, in the heart.^[^ ^336^ ^]^ Notably, IPA has been reported to improve the responsiveness of ICIs across various cancers, including melanoma, breast cancer, and CRC, by modulating the stemness program of CD8^+^ T cells and facilitating the expansion of progenitor exhausted CD8^+^ T cells, which are the main responsive cells to anti‐PD‐1 immunotherapy.^[^ ^95^ ^]^ In summary, IPA is anticipated to confer therapeutic benefits for CVD and cancer, as it not only ameliorates atherosclerosis and HF but also enhances the efficacy of antitumor immunotherapy.

## 7 Cardiovascular Effects of Cancer Therapies

Many cancer therapies are known to have either immediate or long‐term cardiotoxic effects.^[^ ^337^ ^]^ This section briefly reviews the cardiovascular effects associated with chemotherapy, immunotherapy, targeted therapy and radiation therapy (**Table** **5**).^[^ ^338^, ^339^, ^340^, ^341^, ^342^ ^]^

**Table 5:** Chemotherapy, immunotherapy, targeted therapy and radiation therapy induce cardiotoxicity.

Therapy	Clinical manifestations of cardiotoxicity	Prevention and treatment
Chemotherapy		
Anthracyclines (doxorubicin, epirubicin)	Heart failure, palpitations or other signs of an arrhythmia, systolic and/or diastolic dysfunction, elevated troponin or BNP, prolonged QT interval.	Clinical markers to monitor cardiotoxicity (e.g., BNP and troponin I). Echocardiography is recommended before anthracycline treatment, within 12 months after completing treatment, and after a cumulative dose of ≥250 mg m^−2^ of doxorubicin or equivalent, to monitor cardiotoxicity.[^353^ ^]^ Control the medication dosage (e.g., cumulative dose of doxorubicin ≤ 550 mg m^−2^.) Use cardioprotective agents (e.g., ACEI or ARB). Potential therapeutic target for cardiotoxicity prevention: mitochondrial transplantation, mtDNA degradation, cGAS‐STING inhibition, targeting myocardial miR‐216a‐5p to mitigate doxorubicin‐induced cardiotoxicity.^[^ ^347^, ^348^ ^]^
Alkylating agents (cyclophosphamide)	LVD, heart failure, atrial fibrillation, myocarditis, pericarditis, arterial thrombosis.	
Antimetabolites (methotrexate, 5‐fluorouracil)	Chest pain, myocardial ischemia, myocardial infarction, coronary vasospasm, atrial fibrillation, ventricular arrhythmias, myocarditis, pericarditis.	
Taxanes (docetaxel)	Congestive heart failure, myocardial ischemia, arrhythmias, chest tightness and shortness of breath, sinus bradycardia.	
Platinum‐based therapy	Arterial vascular disease, venous thrombo‐embolism, hypertension.	
Immunotherapy		
ICI (PD‐1/PD‐L1 inhibitors, CTLA‐4)	Myocarditis, pericarditis, arrythmia, ventricular dysfunction, arterial vascular disease, cardiomyopathy, valvular disease.	Early detection for cardiotoxicity (e.g., electrocardiogram and echocardiography). Split or fractionated dosing to prevent CRS. Early use of corticosteroid (500‐1000 mg/day, 3‐5 days, gradual tapering over 4‐6 weeks) for ICI‐related myocarditis.^[^ ^357^ ^]^ Use IL‐6 inhibitors (tocilizumab) for CRS.^[^ ^360^ ^]^ Potential therapeutic target for cardiotoxicity prevention: endocrine‐cardiac‐immune pathway for ICI related myocarditis;^[^ ^358^ ^]^ using tumor‐derived extracellular vesicles modified with peptides for ICI‐related cardiotoxicity.^[^ ^359^ ^]^
CAR‐T‐cell	Myocarditis, pericarditis, ventricular dysfunction, arterial vascular disease, cardiomyopathy, valvular disease, CRS related atrial fibrillation.	
Targeted therapy		
HER2 antibody (trastuzumab, pertuzumab)	LVD, heart failure.	Reduce targeted therapy dosage. Use cardioprotective agents (e.g., ACEI, β‐blockers). Potential therapeutic target for cardiotoxicity prevention: using NRG‐1β to avert ponatinib‐induced cardiomyocyte apoptosis.^[^ ^369^ ^]^
VEGF inhibitors (sunitinib, sorafenib)	Hypertension, cardiomyopathy, arterial vascular disease, venous thromboembolism.
ALK inhibitors (crizotinib)	QT prolongation, heart failure.
BCR‐ABL inhibitors (imatinib, ponatinib)	Heart failure, hypertension, arrythmias.
Radiation therapy		
Radiation	Pericardial disease, coronary artery disease, valvular heart disease, heart failure, arrhythmias, subclavian artery stenosis.	Reduce radiation‐exposure dose Potential therapeutic target for cardiotoxicity prevention: statin and angiotensin‐converting enzyme‐inhibitor therapy, anti‐inflammatory and antioxidant therapies (not confirmed in clinical practice).^[^ ^341^ ^]^ More precise radiation targeting to avoid the heart.

Common cancer therapies for cardiotoxicity, along with their management/prevention strategies and recent advances. B‐type natriuretic peptide; LVD, left ventricular dysfunction; PD, programmed cell death; PD‐L, programmed cell death 1 ligand; CTLA, cytotoxic T lymphocyte antigen; ICI, immune checkpoint inhibitor; CRS, cytokine release syndrome; HER, epidermal growth factor receptor; VEGF, vascular endothelial growth factor; NRG, neuregulin.

### 7.1 Chemotherapy

Chemotherapy targets tumor cells by disrupting their energy metabolism and inhibiting their proliferation.^[^ ^343^ ^]^ Anthracyclines, among the most widely applied cytotoxic chemotherapeutic agents, are prominently notorious for their cardiotoxicity, which manifests clinically as LVD, arrhythmia, hypertension, or pericarditis.^[^ ^344^, ^345^ ^]^ The underlying mechanism remains unclear, although anthracycline‐induced ROS generated in iron‐dependent chemical reactions and the inhibition of topoisomerase IIβ are believed to be responsible for cardiotoxicity.^[^ ^346^ ^]^ A recent study revealed that lower mitochondrial numbers in cardiomyocytes correlate with increased susceptibility to doxorubicin‐induced cardiotoxicity via mtDNA leakage and cGAS‐STING pathway activation, which drives inflammation and cardiomyocyte senescence.^[^ ^347^ ^]^ Mitochondrial transplantation, mtDNA degradation, and cGAS‐STING inhibition are proposed as potential strategies to mitigate doxorubicin‐induced cardiotoxicity. In addition, targeting myocardial miR‐216a‐5p or inhibiting the release of harmful exosomes from breast cancer cells could alleviate doxorubicin‐induced cardiotoxicity.^[^ ^348^ ^]^ However, the systemic effects of this application still need to be carefully evaluated. Anthracycline‐induced LVD is both cumulative and dose dependent.^[^ ^349^ ^]^ As a result, the recommended cumulative doses of doxorubicin and daunorubicin should not exceed 550 mg m^−2^. However, no dosage is entirely safe in terms of anthracycline‐induced cardiotoxicity. Even individuals exposed to lower doses of anthracyclines (<250 mg m^−2^) remain susceptible to adverse cardiovascular effects.^[^ ^7^, ^350^ ^]^ Other chemotherapies, such as alkylating agents and platinum‐based therapy, can also induce cytotoxicity and myocardial cell death through various mechanisms, including DNA damage. To prevent severe cardiac events, in addition to reducing the therapeutic dose or stopping therapy, closely monitoring BNP and troponin I and the use of cardioprotective agents such as ACEIs or ARBs are recommended.^[^ ^342^ ^]^

### 7.2 Immunotherapy

Immunotherapy aims to leverage and reactivate antitumor immunity to recognize, restrain, and eliminate cancerous cells through the use of ICIs, adoptive cell therapies, oncolytic viruses, and cancer vaccines.^[^ ^351^ ^]^ ICIs include antibodies that target substrates exploited by tumors to promote immune evasion, such as CTLA‐4, PD‐1 and its ligand (PD‐L1).^[^ ^352^ ^]^ According to the 2022 ESC guidelines on cardio‐oncology, ICIs trigger T‐cell overactivation in noncancerous tissues, which causes immune‐related adverse events (irAEs), particularly life‐threatening cardiovascular complications.^[^ ^353^ ^]^ Among these irAEs, myocarditis is the most commonly described and ranks as the second most common fatal adverse event.^[^ ^352^ ^]^ Recently, ICI‐related myocarditis was reported to be mediated by cytotoxic CD8^+^ T cells, which generate an autoimmune reaction against the cardiac‐specific protein α‐myosin.^[^ ^354^ ^]^ Gasdermin‐E, a tumor suppressor that activates CD8^+^ T cells, has been identified as a key driver of ICI‐related myocarditis through pyroptosis and cGAS‐STING signaling.^[^ ^355^ ^]^ The major adverse cardiac events of ICI‐related myocarditis, such as dynamic ST‐T changes, ventricular arrhythmias, II–III° AV block, and new‐onset wall motion abnormalities, could be predicted by electrocardiogram and echocardiography abnormalities.^[^ ^356^ ^]^ Early high‐dose corticosteroids (500–1000 mg d^−1^) for 3–5 days followed by a gradual taper over 4–6 weeks are recommended by most guidelines for patients with ICI‐associated myocarditis.^[^ ^357^ ^]^ Because ICI treatment reduces serum 17β‐estradiol levels, leading to the downregulation of MANF and HSPA5 in the heart, which exacerbates ICI‐related myocarditis, targeting the endocrine‐cardiac‐immune pathway has become a potential new treatment strategy.^[^ ^358^ ^]^ A newly developed Biomeder technique was reported to target myocardial cells via the use of tumor‐derived extracellular vesicles modified with peptides, which effectively alleviated myocardial damage and preserved antitumor T‐cell activity.^[^ ^359^ ^]^ Chimeric antigen receptor (CAR)‐T cells, a form of adoptive cell therapy, are artificially engineered T cells that can recognize and attack tumor cells. Cytokine release syndrome (CRS), caused by the release of cytokines and chemokines from activated CAR‐T cells, is the most prevalent form of toxicity and a major driver of cardiovascular events following CAR‐T‐cell administration.^[^ ^360^ ^]^ To prevent CRS, split or fractionated dosing is more effective than other infusion schemes in preventing adverse events.^[^ ^360^ ^]^ Tocilizumab, an antibody that targets the IL‐6 receptor and blocks the proinflammatory response caused by IL‐6, has been approved as the first‐line therapy for CRS, either alone or in combination with corticosteroids.^[^ ^360^ ^]^ The clinical approach to manage CRS after CAR‐T‐cell therapy can be categorized into four levels on the basis of symptom severity, as detailed in a previous review.^[^ ^361^ ^]^

### 7.3 Targeted Therapy

The development of targeted therapies aimed at specific molecular targets, such as anti‐HER2 and tyrosine kinase inhibitor (TKI) treatments, has increased. However, approximately 40–45% of patients treated with trastuzumab (a HER2‐targeted therapy) experience a 10% or greater decline in the left ventricular ejection fraction (LVEF).^[^ ^362^ ^]^ HER2 expression in the myocardium, coupled with the disruption of cardiac homeostasis, energy metabolism, and mitochondrial function caused by anti‐HER2 therapy, is considered a contributing factor to LVEF reduction and HF associated with targeted therapy.^[^ ^363^ ^]^ Breast cancer patients receiving trastuzumab treatment after anthracycline treatment benefit from the cardioprotective effects of ACEIs (carvedilol) and β‐blockers (lisinopril).^[^ ^364^ ^]^ Antibody‒drug conjugates (ADCs), such as trastuzumab‐deruxtecan (T‐DXd), have demonstrated significant advantages over standard chemotherapy in terms of efficacy in clinical trials, making them a focal point of drug development.^[^ ^365^ ^]^ Although severe cardiac toxicity appears infrequent, T‐DXd necessitates the same cardiac monitoring protocols as other HER2‐directed therapies.^[^ ^366^ ^]^ TKIs are small molecules that can work intracellularly to inhibit the tyrosine kinase activity of HER2 and other HER family kinases.^[^ ^367^ ^]^ TKIs have detrimental effects on vascular endothelial cells and primarily target cardiomyocytes via ROS and mitochondrial transport chain disruption, resulting in cardiomyocyte contractile dysfunction, autophagy, and apoptosis.^[^ ^367^, ^368^ ^]^ The current management strategies for CVD associated with TKIs include targeted symptomatic treatment, dose adjustment of TKIs, regular monitoring of cardiovascular status, and collaborative efforts between cardiologists and oncologists to ensure comprehensive patient care. Ponatinib, identified as the most cardiotoxic drug among all approved chronic myelogenous leukemia TKIs, induces cardiomyocyte apoptosis through activation of the proapoptotic caspase pathway and inhibits the Akt and ERK signaling pathways, which are vital for cardiomyocyte survival.^[^ ^369^ ^]^ Consequently, enhancing the activation of the Akt and ERK signaling pathways through NRG‐1β has been demonstrated to prevent ponatinib‐induced cardiomyocyte apoptosis.^[^ ^369^ ^]^

### 7.4 Radiation Therapy

Radiation‐induced CVD predominantly occurs after thoracic radiation therapy, specifically in cases of esophageal, breast, and lung cancers, as well as thymoma.^[^ ^344^ ^]^ Radiation therapy induces CVD through multiple mechanisms, including endothelial injury and inflammation, leading to atherosclerosis in coronary arteries, fibrosis and thickening of heart valves and the pericardium, and direct damage to the myocardium and conduction system.^[^ ^370^ ^]^ These pathological changes often manifest years after radiation exposure, contributing to a wide range of cardiovascular complications. Radiation‐induced heart disease is characterized by fibrosis in cardiac tissues triggered by inflammation via the NF‐κB pathway.^[^ ^342^ ^]^ Radiation therapy decreases microvascular density, causing myocyte ischemia and oxidative stress.^[^ ^371^ ^]^ Radiation dose is directly proportional to the rate of adverse cardiovascular events in breast cancer patients, with a 7.4% rise per gray and no identified threshold.^[^ ^372^ ^]^ Modern radiation techniques offer improved precision in delivering smaller radiation doses and incorporating cardiac shielding to limit heart exposure, thereby striving to maintain cancer‐free survival with a reduced risk of cardiac complications. While high‐dose radiation therapy is known to promote atherosclerosis through vascular injury, newly emerging evidence suggests that low to moderate doses may paradoxically reduce cardiovascular risk by decreasing plaque inflammation and size.^[^ ^373^ ^]^ However, this potential benefit requires confirmation in large population‐based studies to establish robust clinical correlations. Future research should prioritize tailored radiotherapy by creating dose constraints for specific cardiac substructures in appropriate patients to minimize radiation‐associated cardiotoxicity.

## 8 Promising Existing Homotherapy for Heteropathy between CVD and Cancer

In addition to recent discoveries concerning the mechanistic intersections of CVD and cancer, some therapies beneficial for both CVD and cancer treatments are gradually being recognized, especially drugs approved by the FDA with mature clinical applications, which we call homotherapy for heteropathy in CVD and cancer, such as antihypertensive medications (ACEIs), α2‐adrenergic receptor (α2‐AR) agonists, digoxin, aspirin, statins, PCSK9 inhibitors, glucose‐lowering drugs (e.g., metformin, GLP‐1 RA and SGLT2i) and drugs used on stents, all of which are mainstream treatments for CVD (**Table** **6**). This homotherapy is highly praised clinically, given the growing number of patients suffering from both CVD and cancer.^[^ ^374^ ^]^ If the same drug is beneficial for both diseases, we can achieve two benefits.

**Table 6:** Homotherapy for heteropathy in CVD and cancer.

Homotherapy	CVD	Cancer
ACEI/ARB	Block RAAS → blood pressure↓→ treat hypertension	Reduce microvascular hydrostatic pressure in tumor → chemotherapeutic drugs delivery^[^ ^375^ ^]^↑ → the risk and mortality of colorectal cancer↓^[^ ^377^, ^378^ ^]^
α2‐AR agonists	Inhibit sympathetic nerve activity → blood pressure↓→ treat hypertension	Enhance anti‐tumor immunity in immunocompetent tumor models, such as colorectal cancer, melanoma^[^ ^380^ ^]^ Increase PD‐1 expression in TILs → synergistic anti‐tumor effect with PD‐1 inhibitors↑^[^ ^380^ ^]^
Digoxin	Inhibit of Na^+^/K^+^ ATPase → myocardial contractility↑→ treat heart failure	Downregulate cell‒cell adhesion in CTC cluster → CTC cluster dissolution → metastatic potential of breast cancer↓^[^ ^381^ ^]^
Aspirin	Inhibit COX‐1 → platelet aggregation↓→ prevent stroke and heart attack^[^ ^384^ ^]^	Downregulate platelets driven pro‐carcinogenesis/metastasis, decrease PD‐1 expression in CD8+T cells and macrophages, modulate DNA mismatch repair and microsatellite instability → all‐cause mortality of 18 cancers↓, particularly in the context of colorectal cancer^[^ ^386^, ^387^ ^]^
Statins	Inhibit HMG‐CoA reductase → cholesterol levels↓→ treat atherosclerosis^[^ ^391^ ^]^	Induce cancer cells apoptosis by promoting BMP2 expression → SMAD4^+^ CRC risk↓^[^ ^394^ ^]^ Modulate gut microbiota → *Lactobacillus reuteri* and their tryptophan catabolite ILA↑→ IL17 signaling↓→ antitumor immunity↑ and facilitate ICI treatment in melanoma^[^ ^396^ ^]^ Target mevalonate pathway → prevent p53‐deficient liver tumors^[^ ^339^ ^]^ Have controversial effects in PDAC^[^ ^401^, ^402^, ^403^ ^]^
PCSK9 inhibitors	Inhibit LDL receptor degradation → LDL‐cholesterol↓→ treat atherosclerosis^[^ ^404^ ^]^	Restore MHC I expression on tumor cell surface→ intratumoral infiltration of CD8^+^T‐cell↑ → synergize anti‐PD‐1 therapy in melanoma, breast and colorectal cancer^[^ ^405^ ^]^ Target LRP1 receptors→ repress metastasis‐promoting genes XAF1 and USP18→ suppress breast cancer metastasis^[^ ^406^ ^]^
Metformin	Target AMPK signaling pathway → glyco‐oxidation↓→ systemic oxidative stress↓→ prevent atherosclerotic CVD^[^ ^408^ ^]^ Target AMPK signaling pathway → cardiomyocyte apoptosis↓→ treat heart failure^[^ ^408^ ^]^	Large cohort trials assess metformin's clinical efficacy as cancer therapeutic remain inconclusive.
Antiproliferative agents for drug‐eluting stents	Commonly used drugs in the coronary stents: mTOR inhibitors (e.g., rapamycin) and Taxol derivatives (e.g., paclitaxel) → antiproliferation of VSMCs → treat atherosclerotic heart disease^[^ ^414^ ^]^	mTOR inhibitor: anticancer therapy for tumors with upregulation of mTOR signaling^[^ ^415^ ^]^ Paclitaxel: chemotherapeutic drug for lung, breast and ovarian cancer^[^ ^415^ ^]^
Anti‐PD‐1/PD‐L1 antibodies	Fc‐binding anti‐PD‐1 mAb captured by FcγRs on myeloid cells → suppress the activated and pro‐inflammatory PD‐1^+^ T cells in atherosclerosis plaque → atherosclerosis plaque area↓^[^ ^421^ ^]^	Interact with PD‐L1 expression on cancer cells and PD‐1 receptor on T cells → T‐cell activation↑→ anti‐tumor immunity↑^[^ ^419^ ^]^
Tipifarnib	Reduce TAC‐induced exosomes → prevent heart from left ventricular dysfunction, hypertrophy and fibrosis → treat TAC pressure‐overload HF^[^ ^430^ ^]^	Disrupt HRAS function → survival of HRAS‐mutant HNSCC patients↑^[^ ^427^ ^]^ Inhibit exosome biogenesis and secretion→ prostate cancer progression↓^[^ ^428^ ^]^

The mechanistic intersections between CVD and cancer highlight the potential for proposing homotherapy. COX‐1, cyclooxygenase‐1; MMR, mismatch repair; TAC, transverse aortic constriction; HNSCC, head and neck squamous cell carcinoma.

### 8.1 ACEIs/ARBs

The tumor vasculature is often in a disordered state with high microvascular hydrostatic pressure and transcapillary fluid movement. This condition, combined with poor lymphatic drainage, leads to high interstitial fluid pressure in the tumor interstitium, which is unfavorable for chemotherapeutic drug delivery.^[^ ^375^ ^]^ Clinical data also support an increased incidence of new‐onset hypertension in cancer patients, especially with chemotherapy exposure.^[^ ^376^ ^]^ Therefore, antihypertensive drugs have been explored as potential homotherapies for cancer, with the aim of normalizing tumor vasculature function. In Cheung et al.’s retrospective study of 187 thousand eligible patients who underwent colonoscopy, the use of ACEIs and ARBs was associated with a lower risk of CRC in a duration‒response manner, with CRC developing less than 3 years after the index colonoscopy (HR 0.78).^[^ ^377^ ^]^ Similarly, on the basis of the SEER‐Medicare database, Balkrishnan et al. reported that the use of antihypertensive drugs, including ACEIs (HR 0.84), β‐blockers (HR 0.87) and thiazide diuretics (HR 0.83), was associated with decreased CRC‐specific mortality.^[^ ^378^ ^]^ However, the anticancer effects of ACEIs may vary depending on the cancer type and drug class. For example, in a population‐based cohort study with 997 thousand hypertensive patients newly treated with ACEIs or ARBs, ACEI use was found to be associated with an increased risk of lung cancer compared with ARB use.^[^ ^379^ ^]^

### 8.2 α2‐AR Agonists

Another antihypertensive medication, α2‐AR agonists, was recently found to upregulate innate and adaptive immune responses in multiple immunocompetent tumor models, such as CRC and melanoma, leading to very strong antitumor activity, even when used as monotherapy.^[^ ^380^ ^]^ Treatment with α2‐AR agonists, such as clonidine, can also lead to increased PD‐1 expression in tumor‐infiltrating lymphocytes, and combining PD‐1 inhibitors and α2‐AR agonists has a strong synergistic antitumor effect.^[^ ^380^ ^]^Encouragingly, α2‐AR agonists, which have been used for decades in humans to lower blood pressure with proven pharmacology and safety profiles, have been shown to have strong potential in antitumor treatment, warranting clinical testing of α2‐AR agonists in cancer patients and suggesting preferential prescription of α2‐AR agonists for patients with hypertensive cancer.

### 8.3 Digoxin

Digoxin, a Na^+^/K^+^ ATPase inhibitor, is used to treat HF. The anticancer effects of digoxin on cancer have been supported by many in vitro and in vivo studies, which have shown that it mainly inhibits tumor growth by causing cell cycle arrest.^[^ ^381^ ^]^ In contrast, data derived from clinical studies demonstrated a significantly increased risk of breast, lung, and colorectal cancer upon digoxin use compared with nonuse.^[^ ^382^ ^]^ Nevertheless, a recent study of the effects of digoxin on circulating tumor cells has been impressive. Circulating tumor cells (CTCs) and CTC clusters, recognized as the primary culprit behind cancer metastasis, exhibit distinct biological characteristics compared with those of cancer cells in primary tumors, rendering conventional anticancer treatment less effective against them. There is still a lack of effective therapies targeting CTCs and CTC clusters. A prospective, open‐label, proof‐of‐concept study in women with metastatic breast cancer demonstrated that digoxin can lead to partial CTC cluster dissolution.^[^ ^383^ ^]^ They reported a mean cluster size reduction of −2.2 cells per cluster upon digoxin treatment among nine metastatic breast cancer patients, and their transcriptome profiling of CTCs also revealed a significant reduction in the expression of cell–cell adhesion genes, meeting the cluster‐dissolution activity.^[^ ^383^ ^]^ As the first‐in‐human proof of principle, this study revealed that digoxin administration at a relatively low dose significantly reduced the CTC cluster size, albeit with mild effects. Future studies are anticipated to employ higher dosages with prolonged treatment durations in expanded patient cohorts to establish robust clinical evidence, supporting the therapeutic application of digoxin in targeting CTC clusters.

### 8.4 Aspirin

Aspirin is one of the oldest and most widely used drugs for the primary and secondary prevention of CVD events, including myocardial infarction and stroke.^[^ ^384^ ^]^ The principal pharmacological action of aspirin is to inhibit the cyclooxygenase (COX) enzyme responsible for the production of prostaglandins (PGs) and thromboxane (TX).^[^ ^385^ ^]^ Recent research highlights the anticancer properties of aspirin on the basis of biological evidence that aspirin regulates cancer‐associated inflammation, platelet‐driven procarcinogenic/metastatic activity, DNA mismatch repair, and microsatellite instability.^[^ ^386^ ^]^ Elwood et al.’s meta‐analysis of 118 observational studies on aspirin and cancer survival reported a 21% reduction in all‐cause mortality across 18 cancers,^[^ ^387^ ^]^ indicating the potential pancancer therapeutic benefits of aspirin. Nevertheless, research on aspirin is relatively rare in the context of CRC. The key downstream molecules regulated by aspirin, PGs and TX promote CRC development and metastasis. Consistently, in a clinical cohort study, the ability of aspirin to lower CRC risk was dependent on COX‐2 expression in tumor tissue.^[^ ^388^ ^]^ In colitis‐associated CRC, aspirin also has immunomodulatory effects on cancer progression through triggering the expression of specialized proresolving mediators (AT‐SPMs) in colonic tissues and downregulating PD‐1 expression in CD8+ T cells and macrophages.^[^ ^389^ ^]^ In addition, aspirin is closely linked to cancer metastasis because of its essential role in regulating platelets. Aspirin has a widespread inhibitory effect on lung metastasis by inhibiting the COX‐1/TXA2 pathway in platelets, thereby reducing platelet aggregation, which promotes a favorable intravascular metastatic niche for cancer cell seeding.^[^ ^390^ ^]^ Although aspirin has unique anticancer effects, especially in CRC, we remain vigilant about the associated side effects, especially bleeding, whether gastrointestinal or intracerebral, which could be a crisis for patients with underlying diseases or for elderly patients.

### 8.5 Statins

Statins, a class of cholesterol‐lowering medications, have been accepted as a cornerstone therapy for the prevention of CVD, especially ASCVD. Clinically, statins are recommended for the primary prevention of CVD, particularly in adults with CVD risk factors, such as dyslipidemia, diabetes, hypertension, or smoking.^[^ ^391^ ^]^ Two decades ago, a case–control study involving 1,953 CRC patients and 2,015 controls reported that statin use for at least 5 years was significantly associated with a decreased relative risk of CRC (odds ratio 0.50).^[^ ^392^ ^]^ A recent study also demonstrated a duration‐dependent relationship between statin use (≥2 years) and a lower risk of CRC morbidity and mortality in patients with inflammatory bowel disease (IBD), a high‐risk population for CRC.^[^ ^393^ ^]^ Several mechanisms through which statins influence CRC risk have been uncovered. In a previous study by Kodach et al.,^[^ ^394^ ^]^ statins were found to induce apoptosis in CRC cells by promoting the expression of bone morphogenetic protein 2 (BMP2). Statin therapy was more effective in CRC cells expressing SMAD4, a central element of the BMP pathway. Using a Netherlands pharmacy database, Ouahoud et al. reported that statin use was associated with a greater risk reduction in the development of SMAD4‐positive CRC (odds ratio 0.64).^[^ ^395^ ^]^ Han et al. reported that statins repress CRC partly by modulating the gut commensal microbiota, particularly *Lactobacillus reuteri*.^[^ ^396^ ^]^ *Lactobacillus reuteri* increases in the mouse gut as a result of elevated microbial tryptophan after statin treatment, and the tryptophan catabolite indole‐3‐lactic acid (ILA) then downregulates IL‐17 signaling to exert anticancer effects.^[^ ^396^ ^]^ Inspiringly, a tryptophan metabolite released by intratumoral *Lactobacillus reuteri* can induce antitumor immunity and facilitate ICI treatment in melanoma,^[^ ^397^ ^]^ indicating the potent benefits of statins in other cancers as well. In addition to CRC, statin use is also associated with a lower risk of several other cancers. Prospective results from 146 thousand participants demonstrated the benefit of statin use on all‐cancer survival.^[^ ^398^ ^]^ The underlying mechanisms are still unclear. Statins reduce cholesterol by targeting 3‐hydroxy‐3‐methyl‐glutaryl (HMG)‐coenzyme A (CoA) reductase (HMGCR), the rate‐limiting step in the mevalonate pathway. Interestingly, recent findings have shown that the mevalonate pathway is a novel downstream target of the famous tumor suppressor p53 in repressing liver cancer, which indicates the therapeutic potential of statins for the prevention of p53‐deficient liver tumors by targeting the mevalonate pathway.^[^ ^399^ ^]^ Nevertheless, Dorsch et al. reported the intricate role of statins in regulating EMT, which is one of the hallmarks of cancer cell metastasis. Their findings showed that while statin‐induced EMT promotes metastatic seeding, it unexpectedly counteracts metastatic foci formation.^[^ ^400^ ^]^ However, we should pay attention to the complexity of the effects of statins in different cancer types and contexts. The clinical application of statins in pancreatic cancer is still relatively controversial.^[^ ^401^, ^402^ ^]^ A recent study demonstrated that, in Trp53^−/−^ PDAC mouse models, genetic or pharmacological inhibition of cholesterol metabolism with statins promoted the formation of basal‐like pancreatic tumors, which are more aggressive.^[^ ^403^ ^]^ Conversely, suppression of the cholesterol biosynthetic pathway in Trp53^+/−^ PDAC mouse models significantly inhibited malignant progression.^[^ ^403^ ^]^ This study indicates that certain patients, particularly those with Trp53‐mutant tumors, may need to reconsider statin use when managing advanced pancreatic cancer. Furthermore, these adverse effects highlight the need for statin‐related population‐based studies to further investigate genotype interactions, thereby enabling a more precise understanding and a more balanced perspective on the role of statins in cancer treatment and prevention.

### 8.6 PCSK9 Inhibitors

In addition to statins, PCSK9 has emerged as a new milestone in cholesterol‐lowering targets. Antibody‐based PCSK9 inhibitors (e.g., alirocumab and evolocumab) have successfully progressed to clinical applications for reducing the risk of ASCVD events, with marked effects on reducing LDL cholesterol levels.^[^ ^404^ ^]^ As a key regulator of cholesterol metabolism, PCSK9 directs the LDL receptor to lysosomal degradation, which is responsible for clearing LDL cholesterol from the circulation, leading to high levels of plasma LDL cholesterol.^[^ ^404^ ^]^ Given that cholesterol is crucial in MHC I molecule recycling, some researchers have discovered that PCSK9 can disrupt the recycling of MHC I molecules to the cell surface by physically associating with MHC I and promoting the lysosome‐mediated degradation of MHC I in tumor cells.^[^ ^405^ ^]^ Interestingly, Liu et al. reported that inhibiting PCSK9 with clinically approved PCSK9‐neutralizing antibodies can significantly increase the intratumoral infiltration of CD8^+^ T cells by restoring MHC I expression on the tumor cell surface and synergize with anti‐PD‐1 therapy to suppress tumor growth in mouse models of melanoma, breast cancer, and colorectal cancer.^[^ ^405^ ^]^ Moreover, a common missense germline variant in PCSK9 (rs562556, V474I) has most recently been found to be associated with reduced survival in multiple breast cancer patient cohorts, and the large Swedish early‐stage breast cancer cohort demonstrated that rs562556 homozygotes represent a 22% risk of distant metastatic relapse compared with nonhomozygotes, who had only a 2% risk.^[^ ^406^ ^]^ Genetic modeling of this gain‐of‐function single‐nucleotide variant in mice supported the crucial role of PCSK9 in promoting breast cancer metastasis, mainly by targeting low‐density lipoprotein receptor‐related protein 1 (LRP1) receptors, which repress the metastasis‐promoting genes XAF1 and USP18.^[^ ^406^ ^]^ Accordingly, antibody‐mediated therapeutic inhibition of PCSK9 robustly suppressed breast cancer metastasis in their study.^[^ ^406^ ^]^ Interestingly, inherited genetic alterations in PCSK9 can govern breast cancer metastasis, indicating that antibody‐based PCSK9 inhibitors are promising homotherapies for breast cancer and CVD. In addition, we should pay attention to the nuanced manipulation of the cholesterol biosynthetic pathway by PCSK9 in cancer cells. In the most recent study of PDAC, PCSK9 was demonstrated to be able to switch cancer cell metabolism between cholesterol import (PCSK9‐low) and synthesis (PCSK9‐high), which dictates the metastatic organ preference of PDAC cells to the liver or lung.^[^ ^407^ ^]^ PCSK9‐low liver‐avid PDAC cells take up LDL cholesterol to activate mTORC1 signaling and produce 24‐HC, leading to reprogramming of the liver microenvironment to promote tumor growth.^[^ ^407^ ^]^ In contrast, PCSK9‐high lung‐avid cells synthesize cholesterol to generate 7‐DHC and 7‐DHD, which can protect them from ferroptosis in the oxygen‐rich microenvironment of the lung.^[^ ^407^ ^]^ This finding reflects the complexity of PCSK9 regulation in cancer progression and indicates that different downstream signaling pathways can be exploited to target PCSK9 in different TME scenarios.

### 8.7 Metformin

Metformin, a well‐known AMPK agonist, has been confirmed to have protective effects on cardiovascular outcomes in individuals with DM2, especially in patients with ASCVD and HF.^[^ ^408^ ^]^ The potential cardioprotective mechanisms of metformin depend on improving myocardial and vascular functions, such as decreasing cardiomyocyte apoptosis, increasing endothelial function or reducing vascular inflammation.^[^ ^409^ ^]^ Due to the role of AMPK as a tumor suppressor, extensive basic research has been conducted to investigate the anticancer biological functions of metformin. Although basic research has yielded positive results, large cohort trials assessing the clinical efficacy of metformin as a cancer therapeutic remain inconclusive.^[^ ^410^ ^]^ In the design of those clinical trials, metformin was assessed in combination with chemotherapy, endocrine therapy or other targeted therapies in most cases.^[^ ^410^ ^]^ Therefore, the role of metformin in cancer treatment and prevention needs further exploration in prospective clinical studies, with a focus on specific populations, such as obese patients, and longer‐term observations. The latest challenge to the role of metformin comes from the emergence of novel glucose‐lowering drugs, such as GLP‐1 RA and SGLT2i, particularly with respect to cardiovascular protection. Multiple large cardiovascular outcome trials have described significant reductions in major cardiovascular events and additional cardiovascular outcomes, such as HF and ASCVD, in patients with DM2.^[^ ^411^ ^]^ Nevertheless, a head‐to‐head comparison of the effects of metformin and SGLT2i on cardiovascular outcomes (NCT03982381) is currently underway.^[^ ^409^ ^]^ Certainly, this has also triggered interest in whether these new glucose‐lowering drugs, such as GLP‐1 RA, have anticancer effects. A recent study of a large cohort of 1.6 million DM2 patients with no prior diagnosis of obesity‐associated cancers revealed a decreased risk of 13 obesity‐associated cancers in patients taking GLP‐1RAs compared with those taking insulin or metformin.^[^ ^412^ ^]^ Another recent study also revealed that GLP‐1 RAs were related to a lower CRC risk in drug‐naïve DM2 patients, with more pronounced effects in obese/overweight patients.^[^ ^413^ ^]^ Until now, relevant evidence for the anticancer effect of GLP‐1RA has remained limited to draw a definite conclusion.

### 8.8 Antiproliferative Agents for Drug‐Eluting Stents

The implantation of drug‐eluting stents (DESs) is the dominant treatment strategy for patients with atherosclerotic heart disease.^[^ ^414^ ^]^ Two classic drugs are commonly used in coronary stents: mTOR inhibitors (e.g., rapamycin) and Taxol derivatives (e.g., paclitaxel), both of which are antiproliferative agents that prevent restenosis.^[^ ^414^ ^]^ Paclitaxel is a widely used chemotherapeutic drug for treating lung, breast and ovarian cancers through its antimitotic effect, whereas mTOR inhibitors (e.g., rapalog) have been developed as cancer therapeutics because of the frequent upregulation of mTOR signaling in human cancer, which leads to rapid proliferation of cancer cells.^[^ ^415^ ^]^ This finding echoes a recent discovery that atherosclerosis is a smooth muscle cell‐driven tumor‐like disease.^[^ ^416^ ^]^

### 8.9 Anti‐PD‐1 Antibodies

The PD‐1 inhibitory receptor, named the “immune checkpoint”, plays a crucial role as a gatekeeper of immune responses.^[^ ^417^ ^]^ Anti‐PD‐1/PD‐L1 monoclonal antibodies (mAbs) have revolutionized cancer treatment in the last decade, and the number of clinical trials related to anti‐PD‐1/PD‐L1 mAbs continues to increase.^[^ ^418^ ^]^ The great success of anti‐PD‐1/PD‐L1 mAbs in cancer treatment is attributed to the principal mechanism by which PD‐L1 expression on cancer cells and interaction with the receptor PD‐1 on T cells lead to the inhibition of T cell activation, which is the main pathway that inhibits antitumor immunity.^[^ ^419^ ^]^ Data from in vivo studies in mice and clinical interventional studies with sequencing results have drawn attention to the critical role of T cells in driving and modifying the pathogenesis of atherosclerosis.^[^ ^420^ ^]^ Surprisingly, based on a retrospective study, Fan et al. reported that anti‐PD‐1 treatment plays a significant role in reducing atherosclerotic plaque areas by targeting activated and proinflammatory PD‐1^+^ T cells in atherosclerotic plaques.^[^ ^421^ ^]^ They then discovered that Fc‐binding anti‐PD‐1 mAbs can be captured by FcγRs on myeloid cells and suppress the activation of these PD‐1^+^ T cells, which was further validated in a prospective patient cohort.^[^ ^421^ ^]^ These findings indicate that T‐cell‐targeting immunotherapy via anti‐PD‐1 mAbs with Fc‐binding capabilities may also serve as an effective treatment for atherosclerosis. Nevertheless, a cautious reservation should be held regarding PD‐1/PD‐L1 as a homotherapeutic target for atherosclerosis treatment. irAEs, particularly those affecting the cardiovascular system, should not be overlooked in ICI treatment, such as anti‐PD‐1/PD‐L1 mAbs. Cardiovascular irAEs, including myocarditis, cardiac dysfunction without myocarditis, arrhythmias, and acute cardiovascular events, have been reported with incidences of 3.1% for ICI monotherapy and 5.8% for ICI combined therapy.^[^ ^422^, ^423^ ^]^ The mortality rate of ICI‐induced myocarditis (25%‐50%) is much higher than that of other types of myocarditis (4%) over a period of follow‐up.^[^ ^422^ ^]^ According to the ESMO guidelines for managing immunotherapy toxicities, when suspected signs or symptoms are detected, other lethal CVDs should be ruled out, and ICIs should be interrupted immediately.^[^ ^424^ ^]^ Therefore, although anti‐PD‐1 treatment may be considered a potential treatment for atherosclerosis, sufficient caution regarding its adverse effects on the cardiovascular system should be taken in clinical practice.

### 8.10 Tipifarnib

Tipifarnib is a highly selective inhibitor of farnesyltransferase that disrupts HRAS function,^[^ ^425^ ^]^ and it has been approved as a novel targeted drug for treating intractable head and neck squamous cell carcinoma (HNSCC). HNSCC, which usually has a high mutation rate of HRAS with poor clinical outcomes, was first proven to respond to tipifarnib.^[^ ^426^ ^]^ The use of tipifarnib can significantly improve the overall survival of HRAS‐mutant HNSCC patients by 10 months.^[^ ^427^ ^]^ Interestingly, tipifarnib also acts as an inhibitor of exosomes by impeding the biogenesis and secretion of exosomes to slow cancer progression.^[^ ^428^ ^]^ Three key enzymes, Alix (ALG‐2‐interacting protein X), nSMase2, and Rab27a (Ras‐associated binding protein 27a), which are involved in exosome biogenesis and secretion, are the main targets of tipifarnib. Exosomes, which are a subset of extracellular vesicles, contribute to a wide range of biological processes in health and disease, including CVD and cancer, especially in terms of crosstalk with the microenvironment and distant organs.^[^ ^429^ ^]^ Leveraging the significant role of tipifarnib in inhibiting exosomes, Mallaredy et al.^[^ ^430^ ^]^ recently reported the potential of tipifarnib in treating a transverse aortic constriction (TAC) pressure‐overload mouse model of HF. These results showed that tipifarnib can not only reduce the TAC‐induced increase in exosome content but also prevent TAC‐induced cardiac hypertrophy, left ventricular dysfunction, and fibrosis in the heart.^[^ ^430^ ^]^Encouragingly, tipifarnib might be used as a novel therapeutic modality for HF in clinical settings.

In summary, there are many therapeutic overlaps between CVD and cancer treatments.^[^ ^431^ ^]^ Homotherapy enables cancer patients with CVD to obtain additional anticancer benefits from regular cardiovascular medications. For CVD patients at high risk of cancer, the recommendation of specific cardiovascular drugs that may have preventive effects against cancer is likely better. To some extent, homotherapy expands the indications for cardiovascular drugs, but their specific dosages in cancer treatment, as well as their compatibility with traditional cancer therapies such as chemotherapy, targeted therapy, and immunotherapy, need further investigation.

## 9 Conclusions and Perspectives

As the global population ages, the incidence of CVD, cancer, and their coexistence is expected to rise. Epidemiologically, more attention should be given to the shared risk factors for CVD and cancer. We hope that morbidity and mortality from both diseases can be simultaneously reduced through lifestyle interventions targeting their shared risk factors. Primary prevention efforts for CVD and cancer should adopt an interdisciplinary approach. In addition to the lifestyles mentioned in this review, other modifiable risk factors that impact both CVD and cancer, such as social determinants, are gradually receiving increasing attention.^[^ ^16^ ^]^ A recent large‐scale study of 10.7 million Chinese individuals revealed that low education levels are the leading factor for all‐cause mortality,^[^ ^432^ ^]^ with CVD and cancer being the primary causes of death. Currently, most research has focused on whether and how lifestyle modifications influence CVD and cancer. More detailed studies are needed to quantify the individual and overall contributions of all these beneficial lifestyle modifications. Moreover, we cannot overlook the impact of these lifestyle modifications on chronic diseases other than CVD and cancer, as many lifestyles beneficial for these conditions also support the prevention and management of numerous other diseases. In the future, epidemiological data are also expected to be refined to address specific cancer types, CVD subtypes, and diverse ethnic groups, necessitating extensive data accumulation in public health.

Research into the mechanisms of CVD and cancer, two major diseases, has increased substantially in recent years. Owing to advancements in genomics, transcriptomics, epigenomics, proteomics, and spatial omics, our understanding of CVD and cancer pathophysiology has deepened significantly. On the basis of the substantial accumulation of research on CVD and cancer, further studies can explore their mechanistic intersections. Since the human body functions as an integrated system, understanding these two diseases through the whole body and the common underlying mechanisms is conducive to providing more accurate individualized treatments for patients. In addition to the mechanistic intersections summarized in this review, other promising mechanisms involved in the coregulation of CVD and cancer, such as the regulation of the central and peripheral nervous systems and common genetic susceptibility, warrant attention. However, at present, there are still gaps in understanding the pathophysiological mechanisms and corresponding clinical interventions involved.

Over the past few decades, the field of cardio‐oncology has emerged as a new clinical frontier but has focused mainly on the cardiovascular side effects caused by cancer treatments. Less emphasis has been placed on developing homotherapy for CVD and cancer. Patients should be regarded holistically. Patients with CVD often require long‐term medications. Thus, we should also be concerned about the impact of these long‐term medications on cancers, for example, promising homotherapy for heteropathy between CVD and cancer. With respect to the interactions between CVD and cancer treatments, more focus is needed on whether these drugs have synergistic or adverse effects. Clinically, when specific treatments are selected, drugs with proven benefits for both diseases should be prioritized. In view of the pathophysiological complexity and treatment challenges of CVD and cancer, more cardio‐oncologists need to be trained to improve patient care. Rather than managing these conditions separately, joint management and patient education for those with or at high risk of both CVD and cancer should be strengthened. Ideal cancer outcomes rely on coexisting cardiovascular health throughout the entire journey of cancer prevention and treatment.

The field of CVD and cancer research is evolving at an accelerated pace, with both challenges to overcome and promising avenues to explore. The scope of cardio‐oncology should be expanded to involve homotherapy, reflecting the growing recognition of shared root causes between CVD and cancer.^[^ ^433^ ^]^ Accumulating research into the intersection between CVD and cancer has already yielded significant insights into cardiovascular and cancer treatment and will continue to shape clinical practice.

## Conflict of Interest

The authors declare no conflict of interest.

## Author Contributions

S.H.X. and Y.J.S. contributed equally to this work. Conceptualization: Q.S.C. and J.J.Z.; Writing—Original Draft: S.H.X. and Y.J.S.; Writing—Review and Editing: S.H.X., Y.J.S., S.H.X., J.W., and M.L.; Visualization: S.H.X. and Y.J.S.; Funding Acquisition: Q.S.C. and J.J.Z.; Supervision: Q.S.C. and J.J.Z. All authors have read and approved the article.
